# Clinical pharmacokinetics and pharmacometabolomics of *Andrographis paniculata* capsules: Bridging drug disposition and metabolic response to precision medicine

**DOI:** 10.1007/s00210-025-04656-0

**Published:** 2025-10-02

**Authors:** Khim Boon Tee, Didi Erwandi Mohamad Haron, Ili Nadhirah Jamil, Wei Lim Chong, Zaril Harza Zakaria, Lee-Ling Lim, Najihah Mohd Hashim, Hasniza Zaman Huri

**Affiliations:** 1 National Pharmaceutical Regulatory Agency, Ministry of Health Malaysia, 46200 Petaling Jaya, Malaysia; 2 Research Services Division, Institute of Research Management & Service, Universiti Malaya, 50603 Kuala Lumpur, Malaysia; 3 Department of Clinical Pharmacy and Pharmacy Practice, Faculty of Pharmacy, Universiti Malaya, 50603 Kuala Lumpur, Malaysia; 4 Precision Medicine and Omics Centre (PrOmiC), Faculty of Pharmacy, Universiti Malaya, 50603 Kuala Lumpur, Malaysia; 5 Department of Medicine, Faculty of Medicine, Universiti Malaya, 50603 Kuala Lumpur, Malaysia; 6 Department of Medicine & Therapeutics, The Chinese University of Hong Kong, Hong Kong SAR, China; 7 Centre of Printable Electronics, Universiti Malaya, 50603 Kuala Lumpur, Malaysia; 8 Department of Pharmaceutical Chemistry, Faculty of Pharmacy, Universiti Malaya, 50603 Kuala Lumpur, Malaysia; 9 Neuroscience Research Group (NeuRG), Faculty of Pharmacy, 50603 Kuala Lumpur, Malaysia; 10 Centre of the Natural Products Research and Drug Discovery, Universiti Malaya, 50603 Kuala Lumpur, Malaysia

**Keywords:** Pharmacometabolomics, Pharmacodynamics, Pharmacokinetics, *Andrographis paniculata*, Precision medicine

## Abstract

*Andrographis paniculata* (Burm. F.) Nees (AP) is a phytomedicinal plant traditionally used for colds, infections, and diabetes in Southeast Asia. The bioactive diterpenoids such as andrographolide, 14-deoxyandrographolide, and neoandrographolide has anti-inflammatory, antimicrobial, and glucose-lowering effects. Although widely used, clinical studies integrating AP’s drug composition with its pharmacometabolomics responses remain limited. This study integrated pharmacokinetics (PK) and pharmacometabolomics (PMx) to understand dose–response relationships and pharmacological effects of orally administered AP capsules (1000 mg and 2000 mg) to 12 healthy volunteers under fasting conditions. Three biomarkers were measured from five AP brands to determine the highest-concentration AP capsule for dosing. PK analysis used 75 plasma samples while PMx analysis involved 96 plasma and urine samples. Liquid chromatography-tandem mass spectrometry (LC–MS/MS) were used for both analyses. Statistical analysis included multivariate analyses (PCA, PLS-DA), followed by peaks-to-pathway analysis via MetaboAnalyst 5.0. Time to reach maximum plasma concentration (Tmax) for andrographolide, 14-deoxyandrographolide, and neoandrographolide was 1.5 h, with maximum plasma concentration (Cmax) of 10.15 ng mL^−1^, 7.02 ng mL^−1^ and 58.45 ng mL^−1^, respectively. At 1000 mg, AP enhanced steroid hormone biosynthesis, while 2000 mg induced broader metabolic shifts, enriching pathways such as biosynthesis of unsaturated fatty acids, alanine/aspartate/glutamate metabolism, and lysine degradation. No free bioactive compounds were detected in urine, indicating metabolism into conjugated forms. Clinical PK guided PMx revealed metabolic responses supporting AP’s potential as a therapeutic agent for inflammation and glucose lowering effect. Further clinical research could optimize dosing and advance AP as a precision medicine candidate.

**Supplementary Information:**

The online version contains supplementary material available at 10.1007/s00210-025-04656-0.

## Introduction

The consumption of herbal products has been increasing since 2014 (Ekor 2014), a trend observed across both developing and developed countries, fueled by their perceived benefits for general health (Tangkiatkumjai et al. 2020). This trend aligns with the growing interest in personalised medicine, which aims to customise treatments based on individual metabolic and genetic profiles. Such an approach also holds promise for enhancing efficacy and safety of complex herbal formulation (Wang et al. 2018). *Andrographis paniculata* (Burm. F.) Nees is one of the herbal medicines that emerged as a potential therapeutic alternative (Intharuksa et al. 2022). *Andrographis paniculata* (AP) commonly known as “King of Bitters”, has a rich history of use in traditional medicine across Asia including China, India, and Southeast Asia. For centuries, AP has been used to address conditions like diabetes, fever, upper respiratory infections, and inflammation, typically through decoctions, pastes, or extracts (Agarwal et al. 2005; Hartanti et al. 2023; Kumar et al. 2021). Its therapeutic value stems from a rich mix of bioactive compounds, particularly diterpenoid lactones such as andrographolide, 14-deoxy-11,12-didehydroandrographolide, andropanoside, 14-deoxyandrographolide, and neoandrographolide, as well as phenolic compounds, flavonoids, and terpenoids (Adiguna et al. 2023; Dai et al. 2019). These compounds have been extensively studied for their pharmacological properties, including antioxidant, anticancer, anti-inflammatory, antihypertensive, and antimicrobial activities, with most evidence derived from animal and in vitro models (Gupta et al. 2021; Hossain et al. 2021).

Preclinical studies in Wistar rats demonstrated AP’s ability to reduce oxidative stress, genotoxic damage, and inflammation, suggesting potential renoprotective effects against nephrotoxicity induced by anti-tuberculosis drugs and advantages in managing acute respiratory infections (Hossain et al. 2021; Sharma et al. 2022). Clinical evidence from a human study further indicates that AP may lower the risk of pneumonia and improve glycemic control in diabetes, reinforcing its role as a promising herbal medicine (Benjaponpitak et al. 2023). However, despite these advances, the pharmacokinetic (PK) and pharmacometabolomic (PMx) profiles of AP are critical for understanding its absorption, distribution, metabolism, excretion, and systemic metabolic responses remain poorly characterized. Much of the existing studies focused on individual bioactive compounds, often at lower doses according to traditional practice, with limited integration of PK and PMx to elucidate dose-dependent metabolic shifts and their implications for therapeutic efficacy and safety (Panossian et al. 2000; Songvut et al. 2023b; Suo et al. 2007).

This gap is particularly significant in the context of precision medicine, where understanding the interplay between drug disposition and metabolic responses can optimize AP’s clinical application and manage the adverse drug reactions. To address this, the present study investigates the PK and PMx profiles of AP capsules administered at 1000 mg and 2000 mg to healthy volunteers under fasting conditions. By analyzing plasma and urine samples using advanced LC–MS/MS and multivariate statistical approaches, we aim to bridge the knowledge gap, characterize AP’s dose–response relationships, and provide insights into its mechanism of action and potential of AP as a precision medicine candidate.

## Methodology

### Identification and quantification of *Andrographispaniculata* capsules

The concentration of three bioactive compounds, namely andrographolide, neoandrographolide and 14-deoxyandrographolide was determined using liquid chromatography Mass Spectrometry (LCMS). Fifteen capsules (3 from each of 5 AP brands) were processed individually: sonicated for 15 min in ethanol, then filtered. Reference standards were prepared in ethanol at: andrographolide (USP Lot 01344, 40.2 mg mL^-1^) with purity of 99.5%, neoandrographolide (ChemFaces Lot CFS201801, 40.2 mg mL^-1^) and 14-deoxyandrographolide (ChemFaces Lot CFS201901, 19.8 mg mL^-1^) with purity of 98% for both.

The bioactive biomarkers were then quantified using LCMS-QTOF 1260 6540 (Agilent Technologies, USA), and the total number of samples analysed using LCMS was tabulated in Table S1. The stationary phase was performed using a guard column (Zorbax-SB-C8 Rapid resolution cartridge) and reverse phase column (Zorbax-SB-Aq 1.8 µm 2.1 × 50 mm) with 0.1% acetic acid in water (A) and methanol (B) as mobile phase. Gradient: 99% B (0–0.5 min), 99 → 1% B (0.5–3 min), 1% B (3–6 min) at 0.6 mL min^-1^, with 2.5 min post-run. The concentration of the calibration curve, quality control samples, and capsule samples were calculated using Agilent MassHunter Quantitative software. The calibration curve was generated using eight concentrations and the concentration of three bioactive compounds was back-calculated using the calibration curve (Table S2 and Figure S1).

### Clinical trial

This sub-study involved analysis of samples from the parent clinical trial, an open-labeled, randomized, cross-over, single-dose oral administration of the investigational medicine conducted after a minimum 10 h fasting. The study was conducted at University Malaya Medical Centre, registered on clinicaltrials.gov (ID: NCT04161404) and approved by Medical Research Ethics Committee, University of Malaya Medical Centre (MEC ID 2018112–6848). The protocol was published (Tee et al. 2022). The investigational product, Shine Hempedu Bumi 500 mg capsules (Batch no: HBC80701H), was the AP capsules selected based on the high content of bioactive compounds according to quantitative analysis in Section "Identification and quantification of *Andrographis paniculata* capsules" and its registration as herbal medicines with the Ministry of Health, Malaysia (Registration No: MAL 20002365 T). Healthy participants who signed the informed consent form and fulfilled the inclusion criteria were dosed with AP 1000 mg and AP 2000 mg. The 1000 mg and 2000 mg doses were selected to compare moderate and higher intake levels, consistent with prior clinical use of AP extracts (600 mg 1.8 gm/day) (Agarwal et al. 2005). These correspond to approximately 53 mg and 106 mg andrographolide daily, which are within the safety margin established in human PK studies reporting tolerance of 180–360 mg/day (Songvut et al. 2023b). Five subjects provided 15 blood samples each (at time points of 0, 0.5, 1, 1.5, 2, 2.5, 3, 3.5, 4, 5, 6, 8, 10, 12, and 24 h post-dose) collected in K2EDTA tubes. After centrifugation (10,000 rpm, 4 °C, 10 min), plasma was stored at −20 °C. A total of 75 plasma samples from five subjects dosed with AP 2000 mg were used for pharmacokinetics analysis, 48 plasma samples and 48 urine samples from six subjects dosed with 1000 mg and 2000 mg AP were used for pharmacometabolomics analysis.

### Laboratory analytical

#### Sample preparation for pharmacokinetics study

Seven calibrators ranging from 1, 2, 5,10, 20, 50, and 100 ng mL^−1^ were prepared. Frozen plasma was thawed, vortexed-mixes, and processed via protein precipitation: 50 µL plasma plus 100 µL 0.01% ammonia in methanol were vortexed (20 s), centrifuged (14800 rpm, 5 min) and 200 µL supernatant was transferred to 2 mL vials with inserts. LC–MS/MS injection volume: 10 µL. The separation used a Phenomenex Gemini-NX C18 column (150 × 2.1 mm, 5 μm) with 0.01% ammonia (A) and acetonitrile/methanol (1:1, B) as mobile phases (0.30 mL min^-1^). The gradient program: 40% B (0 min), linear increase to 95% B (3 min), hold to 5 min, sharp decrease to 10% B (5.01 min), hold to 7 min, return to 40% B (7.01 min), and re-equilibration to 10 min. Mass detection employed an LCMS-8060NX triple quadrupole with heated ESI in negative mode, monitoring specific MRM transitions: andrographolide (349.3 → 331.2/287.1), 14-deoxyandrographolide (331.2 → 108.0/301.1), and neoandrographolide (479.1 → 317.4/161.1).

#### Sample preparation of pharmacometabolomics study for plasma and urine samples

Plasma samples (blank and subject) were thawed from −80 °C and processed using cold protein precipitation (200 μL plasma + 600 μL acetone:acetonitrile:methanol [1:1:1]). After −20 °C incubation (1 h) and centrifugation (10,000 rpm, 4 °C, 10 min), supernatants were filtered (0.22 μm) into LC–MS vials. System suitability test using vendor-provided internal standards ensured instrument performance parameters met acceptance criteria prior to metabolomics analysis. Samples (plasma and urine) were prepared in four batches. Each batch was analyzed separately in positive and negative ionization modes using a global metabolomics approach, resulting in eight analytical batches. Each analytical batch commenced with blank water, blank water spiked with internal standard (IS), and blank plasma or urine (with and without IS), all meeting the acceptance criteria in Figure S6 and Figure S7. Five pooled quality control (PQC) samples were analyzed at batch start, with additional PQCs interspersed after every fourth subject sample to monitor LC–MS system stability. Chromatogram overlays of all PQCs for every batch were assessed for consistency and instrument stability. Overlays of PQCs with subject samples were also evaluated in Figure S8. All eight batches met predefined acceptance criteria prior to data processing. The randomized subjects’ sample sequence table is tabulated in Table S3.

## Results

### Quantitation of bioactive compounds of *Andrographispaniculata* capsules

**Fig. 1:**
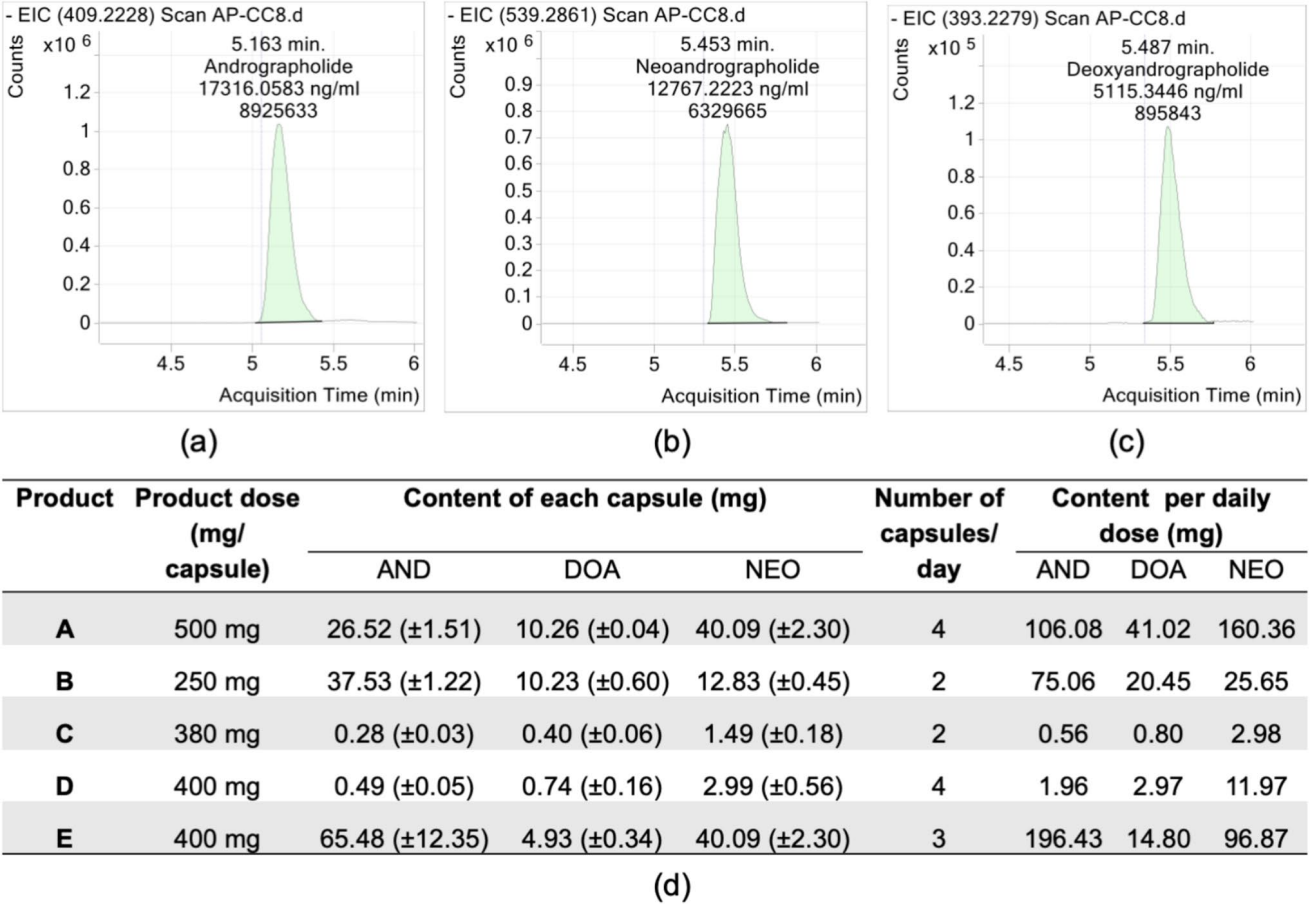
Quantification of major bioactive compounds in AP capsules. Chromatograms of (**a**) andrographolide, (**b**) neoandrographolide, (**c**) 14-deoxyandrographolide, and (**d**) total content of the three bioactive compounds in product A-E based on daily dose recommendations. Chromatograms are shown with X-axis indicating retention time (min) and Y-axis representing detector response. The data presented as mean concentration (ng/mg) ± SD from *n* = 3 capsules per product. Abbreviation: AND, andrographolide; NEO, neoandrographolide; and DOA, 14-deoxyandrographolide

LC–MS/MS analysis quantified three bioactive compounds in AP capsules: andrographolide (5.16 min), neoandrographolide (5.45 min), and 14-deoxyandrographolide (5.49 min) (Fig. 1a-c). Product A (Shine Hempedu Bumi Capsule), contained 26.52 ± 1.51 mg andrographolide, 40.09 ± 1.51 mg neoandrographolide, and 10.26 ± 0.04 mg 14-deoxyandrographolide per capsule. At the recommended daily dose (4 capsules), Product A delivered the highest amounts of neoandrographolide (160.36 mg) and 14-deoxyandrographolide (41.02 mg). Although Product E showed higher andrographolide content (196.43 mg daily), it was excluded because it was an unregistered product in Malaysia. Twelve subjects received Product A (1000 mg and 2000 mg doses) in the clinical trial, with three adverse events reported (all unrelated to treatment). At the administered doses, subjects received approximately 106.7 mg andrographolide, 160.4 mg neoandrographolide, and 41.0 mg 14-deoxyandrographolide (2,000 mg AP), or half these amounts at the 1,000 mg dose (Fig. 1).

### Pharmacokinetics of *Andrographispaniculate* capsules

**Fig. 2:**
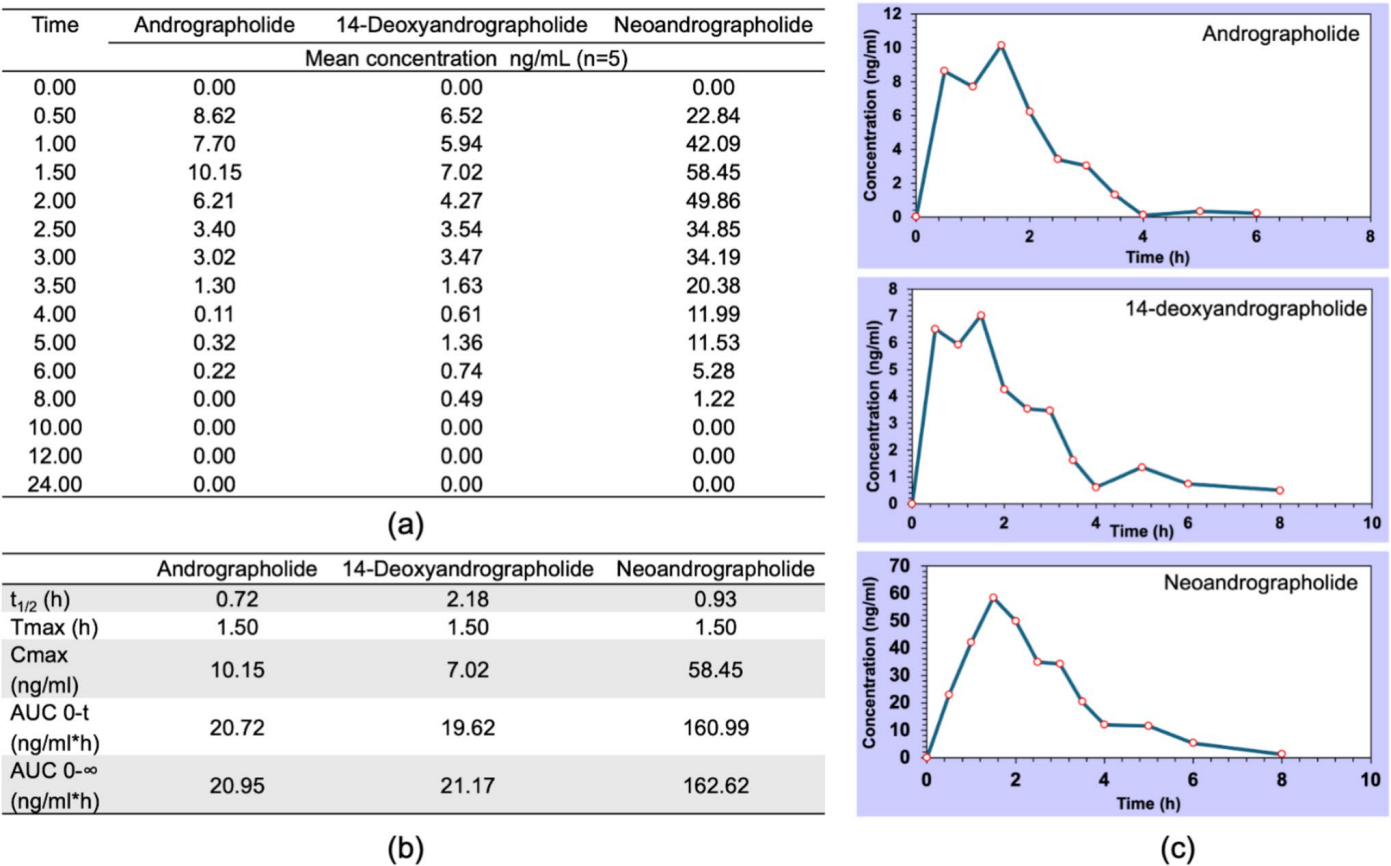
Pharmacokinectic profiles of andrographolide, 14-deoxyandrographolide and neoandrographolide: (**a**) Mean plasma concentration time profile, (**b**) pharmacokinetic parameters for t_1/2_, Tmax, Cmax, AUC_0-t_ and AUC_0-∞_, and (**c**) mean plasma concentration versus time curve for andrographolide, 14-deoxyandrographolide, and neoandrographolide

Pharmacokinetic parameters were determined from plasma samples (5 subjects, 15 time points) analyzed via LC–MS/MS (Shimadzu 8060NX). The MRM transitions for the reference standards showed retention times of approximately 2.6 min for andrographolide and 14-deoxyandrographolide, and 3.1 min for neoandrographolide (Figure S2). Specific MRM transitions for andrographolide, 14-deoxyandrographolide, and neoandrographolide in plasma samples of subjects are illustrated in Figure S3. All compounds showed 1.5 h half-lives, with distinct PK profiles: neoandrographolide demonstrated the highest exposure (Cmax 58.45 ng mL^-1^; AUC0-∞162.62 ng·h mL^-1^), followed by andrographolide (10.15 ng mL^-1^; 20.95 ng·h mL^-1^) and 14-deoxyandrographolide (7.02 ng mL^-1^; 21.17 ng·h mL^-1^). The dose-dependent concentration–time curves, pharmacokinetics profiles (Fig. 2a, b and c) and comprehensive individual PK data (Table S4) provide a foundation for developing personalized AP dosing regimens, supporting its precision medicine potential.

### Pharmacometabolomics of *Andrographispaniculate* capsules

PMx analysis of plasma and urine samples (1000 and 2000 mg AP doses) was performed using MetaboAnalyst 5.0. Principal component analysis (PCA) revealed clustering between pre-dose (T0) and post-dose samples (T2.5-T3.5), with 1000 mg doses separating in negative mode and 2000 mg in positive mode. Partial Least Square Discriminant Analysis (PLS-DA) confirmed these time-dependent trends (Figure S4). Peaks-to-pathway analysis compared metabolic changes pre- vs. post-dose. Some samples were excluded due to technical issues caused by instrument failure and preparation errors.

The sampling time points for PMx analyses were based on the half-life of 1.5 h and intersubject variability in PK. Dose-dependent PMx analyses compared pre-dose (T0) with post-dose time points (T2.5, T3, T3.5) for plasma and pre-dose (U0) with post-dose intervals (0–4 h [U1], 4–8 h [U2], 8–12 h [U3]) for urine, using positive and negative ion mode acquisition. At a 1000 mg dose of AP, steroid hormone biosynthesis and steroid biosynthesis were the most enriched pathways across all treatment times, with an increased number of significant metabolites (*p* < 0.05, enrichment factor > 2.0, Table 1).

**Table 1:** Summary of KEGG pathways following a single oral dose of *Andrographis paniculate* (1000 mg and 2000 mg) at different post-dose time points

1000 mg AP			2000 mg AP		
Human metabolic pathways (KEGG)	Time (hours)	Pathway total/Total hit (*p* < 0.05)	Time (hours)	Human metabolic pathways (KEGG)	Total hit (*p* < 0.05)
Steroid hormone biosynthesis	0 vs 2.5	85/83 (5)	0 vs 2.5	Biosynthesis of unsaturated fatty acids	15 (4)
	0 vs 3.0	85/83 (10)		Alanine, aspartate and glutamate metabolism	9 (3)
	0 vs 3.5	85/83 (20)		Glyoxylate and dicarboxylate metabolism	5 (2)
Steroid biosynthesis	0 vs 2.5	41/41 (1)		Citrate cycle (TCA cycle)	7 (2)
	0 vs 3.0	41/41 (2)		Primary bile acid biosynthesis	32 (2)
	0 vs 3.5	41/41 (3)		Tyrosine metabolism	16 (2)
Primary bile acid biosynthesis	0 vs 2.5	46/32 (1)		Amino sugar and nucleotide sugar metabolism	21 (2)
	0 vs 3.0	46/32 (1)		Steroid biosynthesis	30 (2)
	0 vs 3.5	46/32 (1)		Glycolysis/Gluconeogenesis	12 (1)
Purine metabolism	0 vs 2.5	65/42 (1)		Ascorbate and aldarate metabolism	6 (1)
	0 vs 3.0	65/42 (1)	0 vs 3.0	Biosynthesis of unsaturated fatty acids	16 (4)
	0 vs 3.5	65/42 (1)		Terpenoid backbone biosynthesis	10 (3)
Taurine and hypotaurine metabolism	0 vs 2.5	8/2 (1)		Steroid biosynthesis	40 (6)
	0 vs 3.0	8/2 (1)		Histidine metabolism	7 (2)
	0 vs 3.5	8/2 (1)		Ubiquinone and other terpenoid-quinone biosynthesis	8 (2)
Glycosamino-glycan degradation	0 vs 2.5	21/16 (1)		Lysine degradation	9 (2)
	0 vs 3.0	21/16 (1)		Primary bile acid biosynthesis	32 (4)
	0 vs 3.5	21/16 (1)		Pyrimidine metabolism	15 (2)
Glycosylphosphatidylinositol (GPI)- anchor biosynthesis	0 vs 2.5	11/6 (1)		Tryptophan metabolism	19 (2)
	0 vs 3.0	11/6 (1)		Steroid hormone biosynthesis	78 (7)
	0 vs 3.5	11/6 (1)	0 vs 3.5	Lysine degradation	8 (4)
Arachidonic acid metabolism	0 vs 2.5	35/35 (1)		Ubiquinone and other terpenoid-quinone biosynthesis	8 (3)
	0 vs 3.0	35/35 (1)		Biosynthesis of unsaturated fatty acids	16 (4)
	0 vs 3.5	35/35 (1)		Glycerophospholipid metabolism	3 (2)
Linoleic acid metabolism	0 vs 2.5	4/4 (1)		Alanine, aspartate and glutamate metabolism	12 (3)
	0 vs 3.0	4/4 (1)		Glyoxylate and dicarboxylate metabolism	6 (2)
	0 vs 3.5	4/4 (1)		Primary bile acid biosynthesis	32 (5)
				Histidine metabolism	7 (2)
				Citrate cycle (TCA cycle)	8 (2)
				Cysteine and methionine metabolism	8 (2)

Ranked pathways are enriched using mummichog analysis. Numbers in parentheses indicate significant hits

At 2000 mg, peaks-to-pathway mummichog analysis revealed a shift in regulated pathways, with biosynthesis of unsaturated fatty acids ranking highest at T2.5 and T3, featuring five significant metabolites (stearic acid, oleic acid, arachidonic acid, dihomo-γ-linolenic acid, and docosahexaenoic acid [DHA]). Lysine degradation became prominent at T3.5, with significant hits including saccharopine, allysine, pipecolinic acid, and 4-trimethylammoniobutanoate (*p* < 0.05, Table 1). PCA results for pre- and post-dose urine samples were obtained by performing similar statistical and multivariate analyses of metabolite data (Figure S5). Batch effects were not observed in the normalized datasets for both positive and negative modes at either dose. All urine samples were successfully analyzed, except for samples U13 and U14 for the 1000 mg AP dose in positive mode, which were not captured due to a power supply issue with the LC–MS during the analysis.

**Fig. 3:**
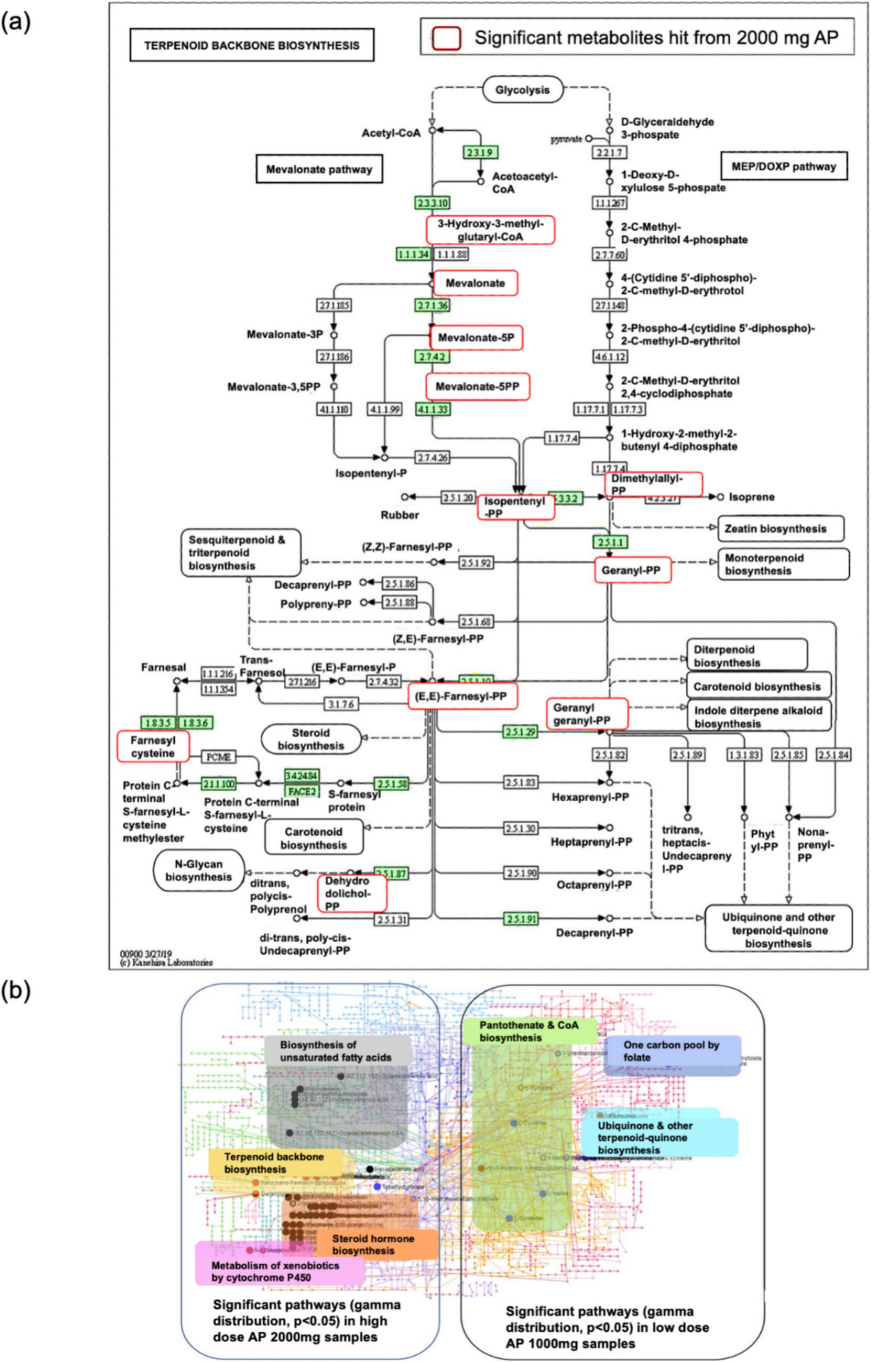
Metabolomic pathway analysis of urine samples following AP administration. (**a**) Terpenoid backbone biosynthesis pathway derived from KEGG, with 11 significant metabolite hits (highlighted in red) detected after 2000 mg AP. (**b**) Predicted human metabolic pathways enriched at 1000 mg and 2000 mg AP when comparing baseline time point (U0) versus post-dose (U1), based on mummichog analysis. The four colored areas in the left box highlight the top 4 pathways enriched at 2000 mg while the right box shows 3 significant pathways enriched at 1000 mg

The trend for significant metabolite hits related to terpenoid backbone biosynthesis was more pronounced at a dose of 2000 mg AP, with a subsequent decrease observed over 12 h (Table 2). The 11 significant metabolite hits were matched with the Kyoto Encyclopaedia Genes and Genome (KEGG) derived terpenoid backbone biosynthesis through the mevalonate pathway (Fig. 3a). The four coloured areas in the left box of Fig. 3b represent the top four pathways identified from 2000 mg AP in urine samples.

**Table 2:** Comparison of total compounds in pathway, total metabolites hit, and number of significant metabolites hit for 2000 mg AP and 1000 mg AP in urine

Human metabolic pathways	Total compounds in pathway/Total metabolites hits (Number of significant metabolite hit, *p* < 0.05)					
	2000 mg AP			1000 mg AP		
	U0 vs U1	U0 vs U2	U0 vs U3	U0 vs U1	U0 vs U2	U0 vs U3
Terpenoid backbone biosynthesis	15/11 (11)	15/12 (9)	15/12 (4)	15/3 (1)	-	-
Steroid hormone biosynthesis	85/83 (68)	85/82 (37)	85/82 (48)	85/35 (14)	85/35 (7)	85/76 (5)
Drug metabolism-cytochrome P450	43/29 (29)	43/37 (21)	43/21 (21)	43/18 (5)	43/18 (4)	-
Metabolism of xenobiotics by cytochrome P450	68/34 (24)	68/32 (20)	68/36 (16)	68/17 (6)	68/21 (4)	68/32 (3)
Biosynthesis of unsaturated fatty acids	34/10 (9)	34/9 (3)	34/15 (9)	34/4 (2)	34/4 (1)	-
Pantothenate and CoA biosynthesis	17/11 (7)	17/10 (3)	17/10 (3)	17/6 (4)	17/6 (2)	17/8 (1)
One carbon pool by folate	9/4 (3)	9/4 (3)	9/4 (1)	9/2 (2)	9/2 (2)	9/6 (3)
Ubiquinone and other terpenoid-quinone biosynthesis	9/7 (4)	9/7 (5)	9/8 (3)	9/2 (1)	-	9/7 (2)

Numbers in parentheses indicate metabolite hits

## Discussion

### Bioactive compounds variability in *Andrographispaniculata* capsules

Prior to pharmacokinetic profiling, quantitative analysis of three bioactive diterpenoids, andrographolide, 14-deoxyandrographolide, and neoandrographolide in AP capsules revealed significant inter-product variability. While literature consistently identifies andrographolide as the predominant diterpenoid in AP extracts (Adam et al. 2023; Adiguna et al. 2023; Cheung et al. 2001), our analysis demonstrated an atypical profile, with neoandrographolide exhibiting the highest concentration (1.3% w/w), followed by andrographolide (0.96%) and 14-deoxyandrographolide (0.34%). This contrasts with recent findings by (Songvut et al. 2023a) who reported substantially higher andrographolide content (3.76%) in GMP-certified aqueous AP extracts, alongside neoandrographolide (0.98%) and 14-deoxyandrographolide (0.56%). Notably, while unregistered Product E contained a pharmacologically relevant andrographolide dose (196.43 mg daily), but it is non-compliance with Malaysian regulatory standards (NPRA, 2008) and imbalanced diterpenoid profile precluded clinical evaluation. These findings suggest a critical need for standardized AP formulations with verified diterpenoid ratios, a prerequisite quality measures for ensuring reproducible pharmacokinetics and therapeutic effects. Such standardization is particularly vital for precision medicine applications, where inter-individual metabolic responses depend on consistent phytochemical composition.

### Pharmacokinetics profiles of bioactive compounds *Andrographispaniculata*

PK properties of bioactive compounds AP (andrographolide, neoandrographolide, and 14-deoxyandrographolide) provide insights into their absorption, distribution, and metabolism. In this study, the time to reach maximum plasma concentration (Tmax) was 1.5 h, longer than the previously reported Tmax values 0.8–1.0 h for a 60 mg of andrographolide equivalent dose (Songvut et al. 2023b). This discrepancy may be attributed to the higher dose used here (106.68 mg andrographolide in 2000 mg AP) or differences in formulation (capsule vs. aqueous extract), potentially due to slower dissolution or increased biotransformation into glucuronide and sulfate conjugates. These conjugates exhibit longer T_1/2_ and higher maximum plasma concentrations (Cmax) (Songvut et al. 2023b). In contrast, a Tmax of 1.5–2 h was reported for a 20 mg andrographolide equivalent dose (Panossian et al. 2000), aligning with our findings and suggesting that formulation or batch consistency may influence bioavailability (Alolga et al. 2015). These dose-dependent PK profiles (e.g., Cmax of 10.15–58.45 ng mL^−1^, AUC0-∞ of 20.95–162.62 ng·h mL^−1^) lay the foundation for developing personalized AP dosing strategies in precision medicine, optimizing efficacy for inflammation and metabolic disorders while minimizing interactions with conventional drugs.

Absorption and the corresponding maximum plasma concentration (Cmax) in the bloodstream have been studied in the context of poly-pharmacokinetics, particularly in Chinese herbal medicine involving healthy volunteers (Xie et al. 2018). This approach allows the investigation of the temporal variations in drug concentration of the targeted bioactive compounds, providing insights into their optimal binding to target sites and subsequent pharmacodynamic effects. In this bioavailability study, the Tmax for andrographolide, neoandrographolide, and 14-deoxyandrographolide in healthy volunteers was 1.5 h, longer than the previously reported Tmax values of 0.8 h (Cmax = 72.1 ± 28.7 ng mL^−1^), 0.9 h (Cmax = 24.4 ± 5.6 ng mL^−1^), and 1.0 h (Cmax = 4.2 ± 3.4 ng mL^−1^), respectively, following the administration of an AP dose corresponding to 60 mg of andrographolide (Songvut et al. 2023b). Nevertheless, the dose of andrographolide used in this study is relatively high, equivalent to 106.68 mg per 2000 mg of AP. In comparison, a PK study conducted by Panossian and colleagues (Panossian et al. 2000) in a randomized, single-dose design of AP (equivalent to 20 mg andrographolide, a dose comparable to the 2000 mg AP used here) reported an average of Tmax of 1.5–2 h, which is longer than that reported by Songvut and co-workers (Songvut et al. 2023b). Variations in herbal medicines formulations or batch-to-batch consistency may further affect the Tmax and bioavailability (Alolga et al. 2015). It was hypothesized that andrographolide, neoandrographolide, and 14-deoxyandrographolide undergo biotransformation into conjugated glucuronide and sulfate metabolites, potentially improving bioabsorption and systemic circulation. Compared to their parent compounds, these conjugated metabolites exhibit longer T_1/2_ and Tmax, as well as higher Cmax values (Songvut et al. 2023a, b). In a related PK study, major compounds of AP were renally excreted within 32 h after administration (Songvut et al. 2023b). The observed half-life range of 0.5 to 2 h demonstrated considerable intersubject variability; therefore, individual pharmacokinetic data may be critical for selecting accurate sampling time points to analyze metabolic pathways, which could subsequently reveal potential efficacy or adverse drug reactions.

### Pharmacometabolomics insights: Dose-dependent metabolic changes

PMx analysis revealed dose-dependent metabolic shifts in AP, providing critical insights into its mechanism of action and therapeutic potential for precision medicine. Computational tools such as mummichog, which identifies regulated human metabolic pathways using the KEGG database, speed up the prediction of functional activity from untargeted metabolites (Chong and Xia 2020; Li et al. 2013; Zhou et al. 2020). At a 1000 mg dose (equivalent to 9.64 mg andrographolide), no significant pathway changes were observed between pre-dose (T0) and post-dose time points (T2.5, T3, T3.5), as determined by mummichog analysis using the KEGG database (*p* > 0.05). However, steroid hormone biosynthesis and steroid biosynthesis emerged as the most enriched pathways, with a significant increase in metabolite hits (*p* < 0.05, enrichment factor > 2.0, Table 2). This enrichment, involving key steroids like cortisol, suggests that 1000 mg AP modulates adrenal and gonadal steroid production, potentially reducing inflammation by inhibiting the expression of steroidogenic acute regulatory protein (StAR) and cytochrome P450scc (CYP11A1), as demonstrated in lipopolysaccharide (LPS)-induced inflammatory rat models (Gupta et al. 2020; Yuan et al. 2022). Cortisol’s role in cancer progression (e.g., ovarian steroidogenesis, prostate cancer), stress response, and neuroactive ligand-receptor interactions further highlights AP’s relevance for managing chronic inflammatory and metabolic conditions (Knezevic et al. 2023; Sapse 1997; Stewart 1999). These findings indicate that 1000 mg AP may exert subtle, targeted anti-inflammatory effects suitable for mild conditions, but insufficient for broader metabolic modulation, limiting its utility in precision medicine at this dose.

In contrast, doubling the dose to 2000 mg elicited distinct pharmacodynamic changes, significantly enriching a broader range of metabolic pathways (*p* < 0.01, enrichment factor > 2.5, Table 2). At T2.5 and T3, the biosynthesis of unsaturated fatty acids ranked highest, with five significant metabolites, stearic acid, oleic acid, arachidonic acid, dihomo-γ-linolenic acid (DGLA), and docosahexaenoic acid (DHA) identified (*p* < 0.01). This pathway’s enrichment, linked to omega-3 fatty acid production, supports AP’s traditional use in inflammation and type 2 diabetes (T2D), as omega-3 fatty acids modulate inflammation via eicosanoid pathways, counteracting insulin resistance and arthritis (Asefy et al. 2021; Pérez-Martínez et al. 2020; Shiels et al. 2022). DGLA, in particular, differentiates healthy and inflamed tissues, with abnormally low levels associated with cardiovascular diseases and atopic dermatitis, and elevated levels linked to T2D. The stability of significant metabolite numbers from T2.5 to T3.5 suggests that unsaturated fatty acid metabolism peaks early, reflecting rapid absorption and bioactivity of AP’s diterpenoids at 2000 mg, critical for precision medicine applications targeting chronic inflammation (Kanda et al. 2021; Nilsen et al. 2021; Ouchi et al. 2017; Simon et al. 2014).

At T3.5, lysine degradation became prominent, with significant hits including saccharopine, allysine, pipecolic acid, and 4-trimethylammoniobutanoate (*p* < 0.05, fold change > 1.5). This delayed enrichment, following the pipecolic acid and saccharopine pathways, aligns with findings of Crowther and co-workers (Crowther et al. 2019), suggesting AP’s potential neurometabolic effects and warranting further investigation into its role in neurometabolic disorders. Additional pathways enriched at 2000 mg, such as alanine/aspartate/glutamate metabolism and glycolysis/gluconeogenesis, indicate broader metabolic regulation. The activation of glycolysis/gluconeogenesis d (*p* < 0.05) is linked to energy production and immune function during inflammation, potentially impacting Alzheimer’s disease and T2D risk (Raj et al. 2021; Yan et al. 2020). Amino sugar and nucleotide sugar metabolism, peaking at T2.5 (*p* < 0.01), further supports AP’s antidiabetic potential, as glucosamine’s anti-inflammatory and antioxidant properties reduce cardiovascular risk and T2D incidence (Ma et al. 2019, 2020; Xu et al. 2022).

Collectively, these enriched pathways fall into functional clusters: lipid metabolism (unsaturated fatty acids, steroids), amino acids metabolism (lysine, arginine/proline, alanine/aspartate/glutamate), nucleotide metabolism (purine, amino sugar and nucleotide sugar), and energy metabolism (glycolysis/gluconeogenesis). Rather than isolated changes, the coordinated pathway changes suggest that AP induces systemic metabolic modulation. This interpretation is consistent with preclinical findings that andrographolide upregulates the PI3K/AKT/eNOS signaling axis that promote production of NO and vascular protection in human endothelial cells (Duan et al. 2019), improves liver health by lowering blood glucose, inflammation, and lipid accumulation in high-fat-diet mice (Hu et al. 2025), and regulate purine catabolism and uric acid homeostasis in a rat model (Rahmi et al. 2022).

These dose-dependent PMx shifts highlight AP’s therapeutic versatility, with 2000 mg inducing broader, more potent metabolic responses than 1000 mg. For precision medicine, these findings enable individualized dosing strategies, where metabolic profiling can identify patients likely to benefit from AP for inflammation or diabetes, optimizing efficacy while minimizing risks like drug interactions or metabolic dysregulation. However, variability in pathway responses across individuals, as suggested by our small cohort, underscores the need for larger, more diverse studies to validate these biomarkers for personalized AP therapy. Although AP is known to possess multiple bioactive compounds, its precise mechanism of action has yet to be fully investigated. Nevertheless, extensive preclinical studies of its pharmacological features have been extensively conducted for andrographolide in the pre-clinical phase whereby it shows efficacy in suppressing inflammation processes, lowering glucose and cholesterol levels as well as providing neuroprotection against cell damage.

In addition to metabolic effects, AP also acts though inflammatory signalling pathways, further supporting the observed metabolomic changes. One mechanism underlying anti-inflammatory effects of andrographolide involves the nuclear factor-κB (NF-κB) signalling pathway. Andrographolide acts as an NF-κB inhibitor by reducing the NF-κB binding activity or deactivating the NF-κB (Cai et al. 2022). Additional pathways for andrographolide’s anti-inflammatory effects include the Janus tyrosine-protein kinase (JAK)/signal transducer and activator of transcription pathway and the T cell receptor signalling pathway (Cai et al. 2022). A network pharmacology study demonstrated IL-6, VEGFA, PTGST2, TNF-α, and MMP-9 are the target genes modulated by AP, significantly reducing their expression to manage inflammatory responses (Zhu et al. 2021). While preclinical data are promising, clinical efficacy of AP remains to be fully established in clinical studies.

Pharmacometabolomics is increasingly recognized as a translational platform for precision medicine, capable of capturing interindividual variability and identifying metabolic signatures predictive of drug response and safety outcomes (Kaddurah-Daouk et al. 2015; Burt and Nandal 2016). Recent reviews highlight its potential to guide drug disposition, efficacy, and toxicity (Trevor et al. 2024), and metabolome wide association studies demonstrate its value in mapping human drug response and stratifying patients for optimized therapy (Schmidt et al. 2021). In this study, dose dependent pharmacometabolomic changes were demonstrated in healthy volunteers, but the absence of patient heterogeneity and treatment response data limits immediate clinical relevance. We therefore present the work as proof of concept. While exploratory, our findings provide an initial step toward applying pharmacometabolomic insights within precision medicine frameworks, which will require validation in larger patient based studies.

### Urinary metabolite analysis and excretion patterns

Urinary PMx analysis detected no free andrographolide, neoandrographolide, or 14-deoxyandrographolide at either dose. It is consistent with a clinical trial conducted on healthy volunteers that identified four andrographolide sulfur conjugates (Cui et al. 2004) and seven andrographolide glucuronide conjugates (Cui et al. 2005), as well as a rodent study (Levita et al. 2017). The enriched diterpenoid backbone biosynthesis pathway at 2000 mg, which decreased over 12 h, suggests that AP’s diterpenoids (e.g., geranylgeranyl-PP) are metabolised via the mevalonate pathway, with polar metabolites such as mevalonic acid excreted in urine (Buhaescu and Izzedine 2007). Geranylgeranyl-PP was a significant compound identified at the 1000 mg dose. This metabolic clearance pattern emphasizes the importance of individualized monitoring in precision medicine to ensure safety and efficacy, particularly in patients with impaired renal function or those taking concomitant medications.

The present study involved 12 healthy volunteers, which limits statistical power and generalizability. While this sample size aligns with established methodological recommendations for pilot investigations that are designed to assess feasibility and estimate variance rather than provide definitive hypothesis testing (Julious 2005), it should be considered as preliminary and hypothesis generating. Larger and more diverse cohorts will be needed to validate and extend these findings. Subsequent Phase II trials should specifically address AP-drug interactions with common metabolic therapies, and clinical implementation of our pharmacometabolomic biomarkers to enable truly personalized herbal medicine approaches.

## Conclusion

In conclusion, integrated PK-PMx analysis reveals that standardised AP establishes consistent linear pharmacokinetics (Tmax = 1.5 h) for andrographolide, 14-deoxyandrographolide and neoandrographolide, with 2000 mg administration eliciting significant dose-dependent pharmacometabolomics perturbations, including enriched biosynthesis of unsaturated fatty acids, lysine degradation, and steroid metabolism. These correlation position AP as a promising therapeutic candidate for metabolic disorders, particularly diabetes mellitus, while providing a scientific framework for precision dosing optimization.

## Supplementary Information

Below is the link to the electronic supplementary material.

Supplementary file1 (DOCX 2.72 MB)

Supplementary file2 (PDF 49 KB)

## Data Availability

All relevant metabolomics data are prodived in the Supplementary Information.

## Declarations

### Clinical trial registration

The study is registered on ClinicalTrials.gov (ID: NCT04161404) on 13.11.2019 and in the National Medical Research Registry (NMRR ID: NMRR-18–2907-41284).
