# Supplementary material for: Clinical pharmacokinetics and pharmacometabolomics of Andrographis paniculata capsules: Bridging drug disposition and metabolic response to precision medicine
Source: Naunyn Schmiedebergs Arch Pharmacol. 2025 Oct 2;399(3):3561–73. doi: 10.1007/s00210-025-04656-0 (PMC12935787; doi:10.1007/s00210-025-04656-0)
Supplement: Supplementary file 1 — Supplementary file1 (DOCX 2.72 MB) [file 210_2025_4656_MOESM1_ESM.docx]

Supplementary materials and figures for

**Clinical Pharmacokinetics and pharmacometabolomics of *Andrographis paniculata* capsules: Bridging Drug Disposition and Metabolic Response**

Khim Boon Tee^a^, Didi Erwandi Mohamad Haron^b^, Ili Jamil^c,d^, Wei Lim Chong^c,d^ , Zaril Harza Zakaria^a^, Lee-Ling Lim^e,f,g^, Najiah Mohd Hashim^h,i,j^, Hasniza Zaman Huri^c,d*^

^a^National Pharmaceutical Regulatory Agency, Ministry of Health Malaysia, Petaling Jaya 46200, Malaysia

^b^Research Services Division, Institute of Research Management & Service, University of Malaya, Kuala Lumpur 50603, Malaysia

^c^Department of Clinical Pharmacy and Pharmacy Practice, Faculty of Pharmacy, Universiti Malaya, Kuala Lumpur 50603, Malaysia

^d^Precision Medicine and Omics Centre (PrOmiC), Faculty of Pharmacy, Universiti Malaya,
 Kuala Lumpur 50603, Malaysia

^e^Department of Medicine, Faculty of Medicine, University of Malaya, Kuala Lumpur, Malaysia

^f^Department of Medicine & Therapeutics, The Chinese University of Hong Kong, Kuala Lumpur, Malaysia

^g^Department of Medicine & Therapeutics, The Chinese University of Hong Kong, Hong Kong SAR, China

^h^Department of Pharmaceutical Chemistry, Faculty of Pharmacy, Universiti Malaya, Kuala Lumpur 50603, Malaysia

^i^Neuroscience Research Group (NeuRG), Faculty of Pharmacy, 50603 Kuala Lumpur

^j^Centre of the Natural Products Research and Drug Discovery, Universiti Malaya.

*Corresponding Author:

Hasniza Zaman Huri, Department of Clinical Pharmacy and Pharmacy Practice, Faculty of Pharmacy, Universiti Malaya, 50603, Kuala Lumpur, Malaysia.

Email: hasnizazh@um.edu.my


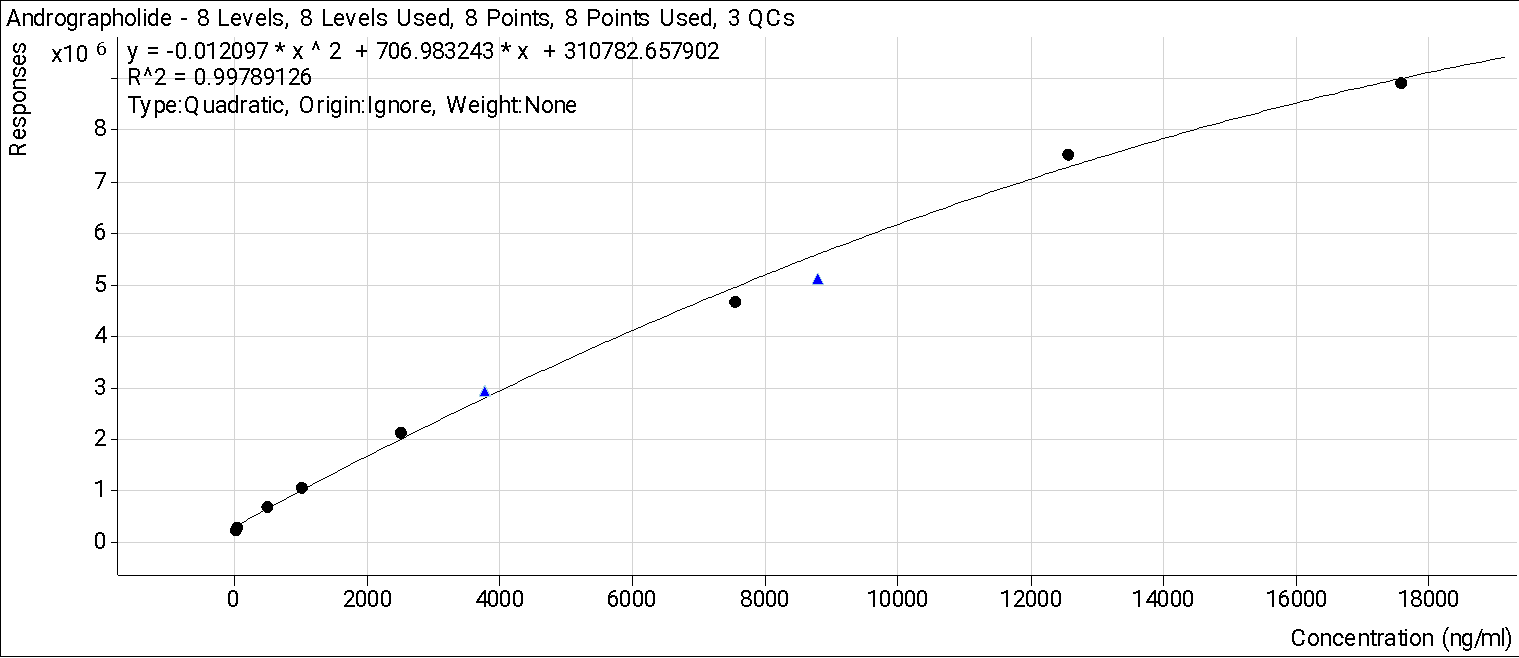


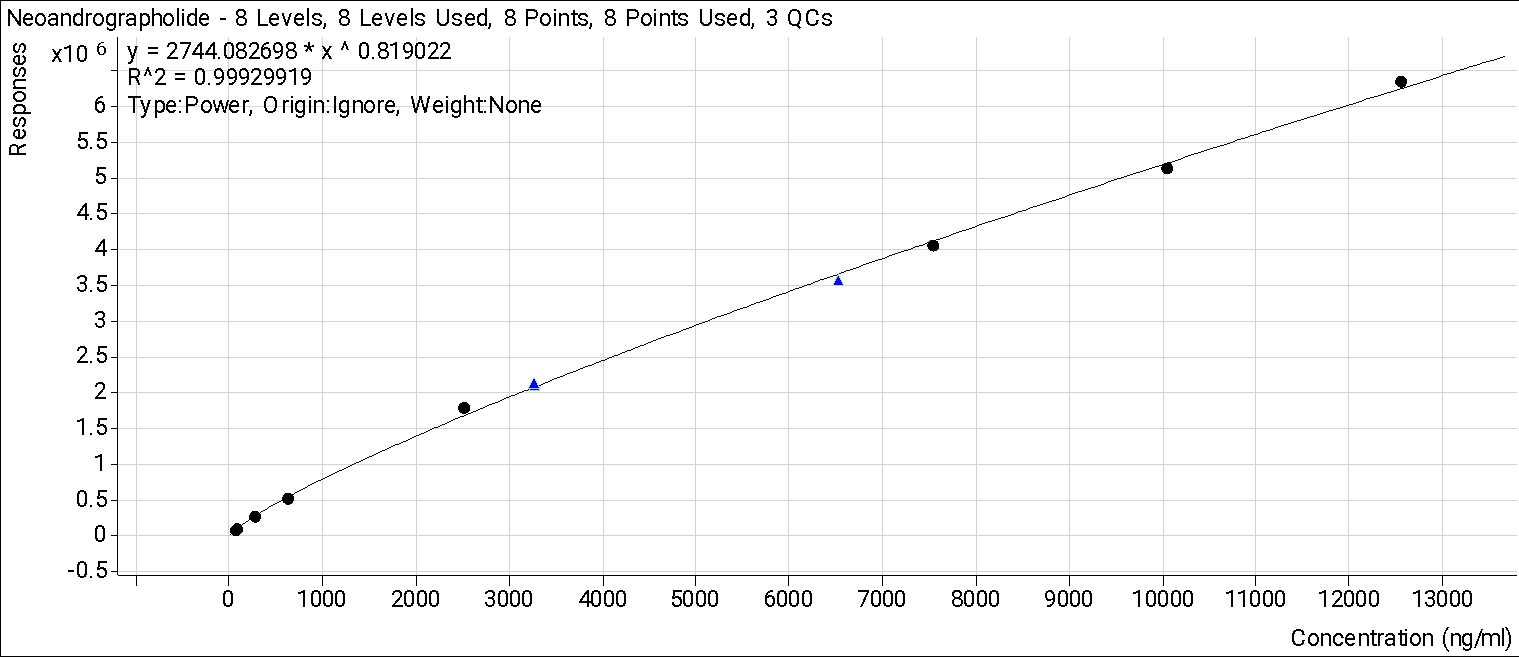


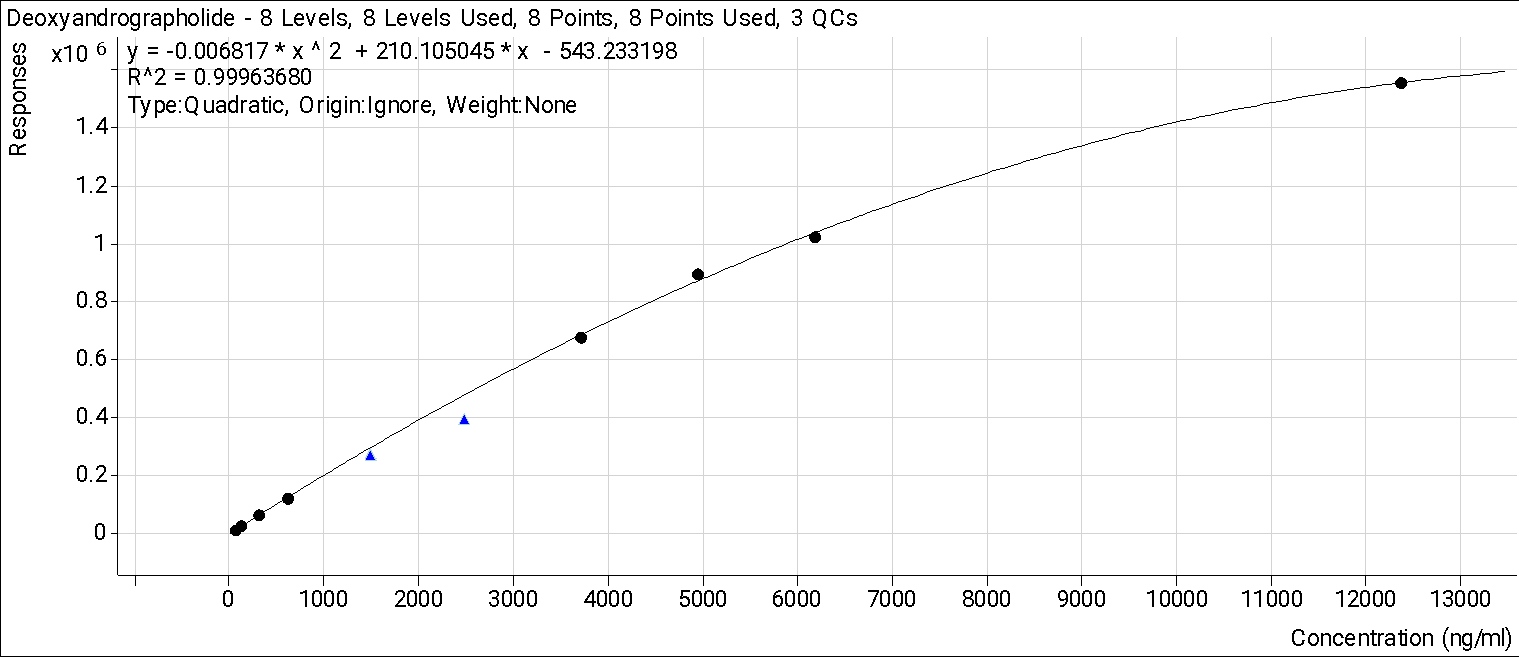


Figure S1. Calibration curves for three bioactive biomarkers (a) andrographolide, (b) neoandrographolide, and (c) 14-deoxyandrographolide in Andrographis paniculata capsules.


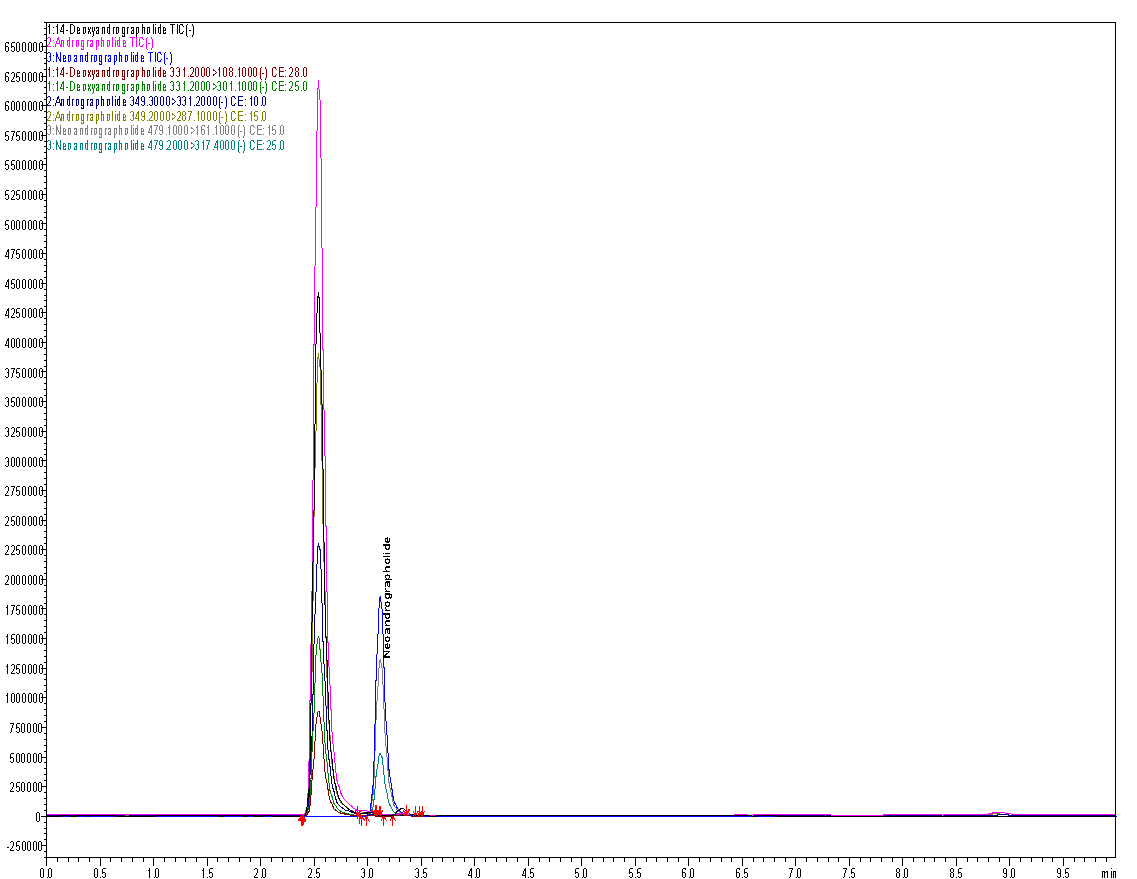


Figure S2. Multiple Reaction Monitoring (MRM) transitions for reference standards of andrographolide, neoandrographolide, and 14-deoxyandrographolide in Plasma.



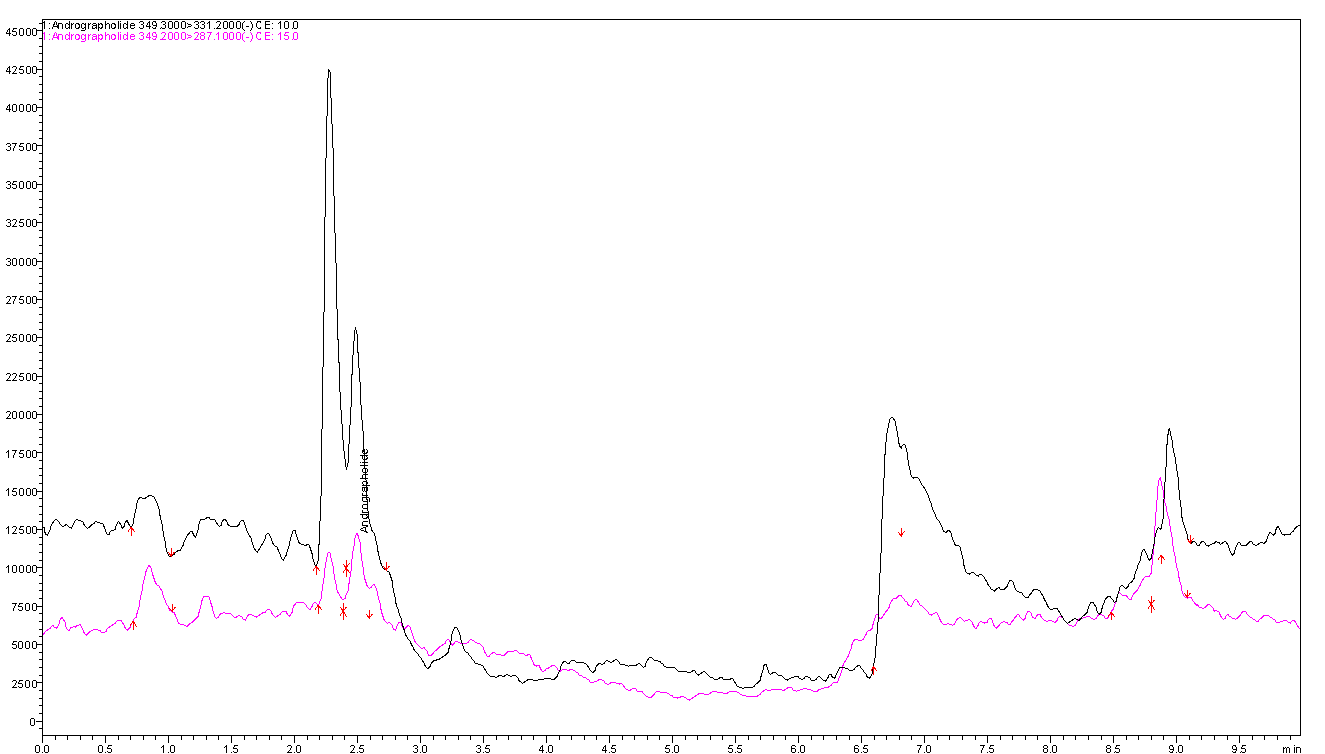



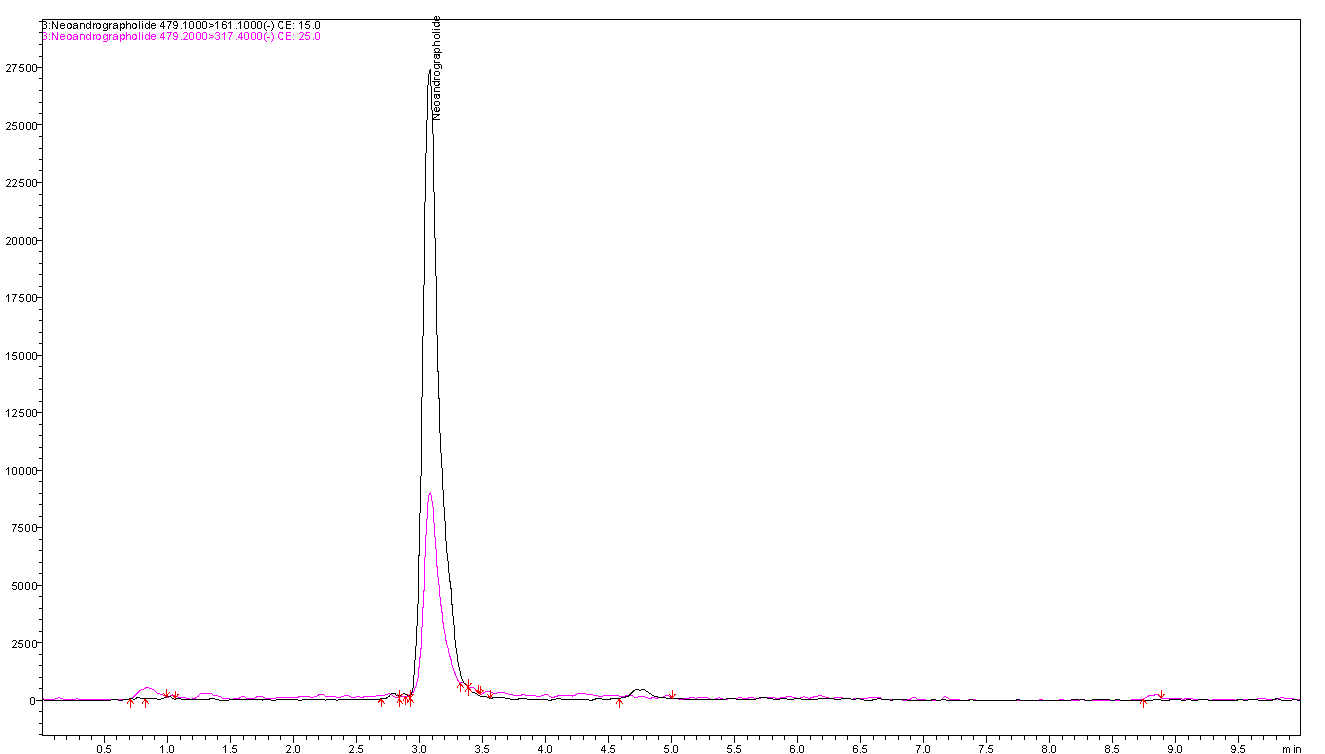


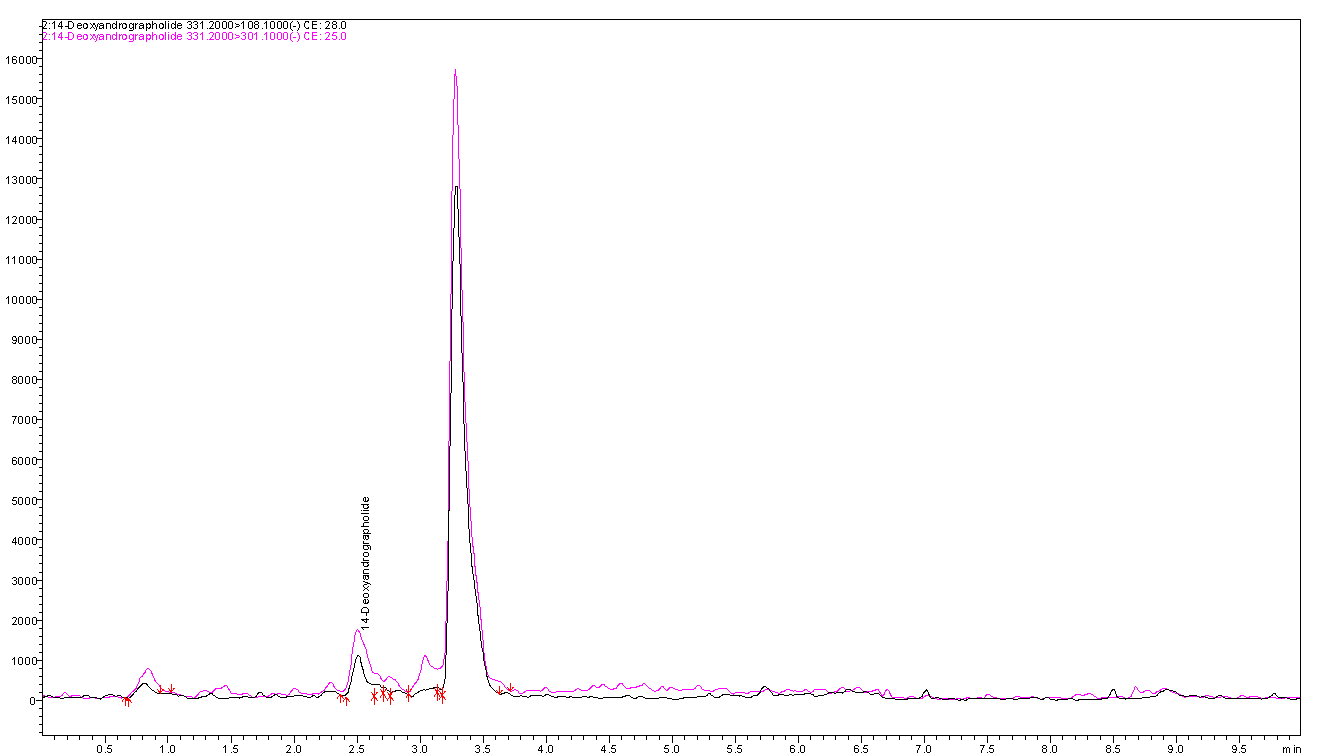


Figure S3. Multiple Reaction Monitoring (MRM) Transitions for (a) Andrographolide, (b) Neoandrographolide, and (c) 14-Deoxyandrographolide in Subject Plasma Samples. MRM parameters (parent ion → daughter ion transitions, retention times) in plasma samples from subjects administered 1000 mg and 2000 mg Andrographis paniculata capsules.



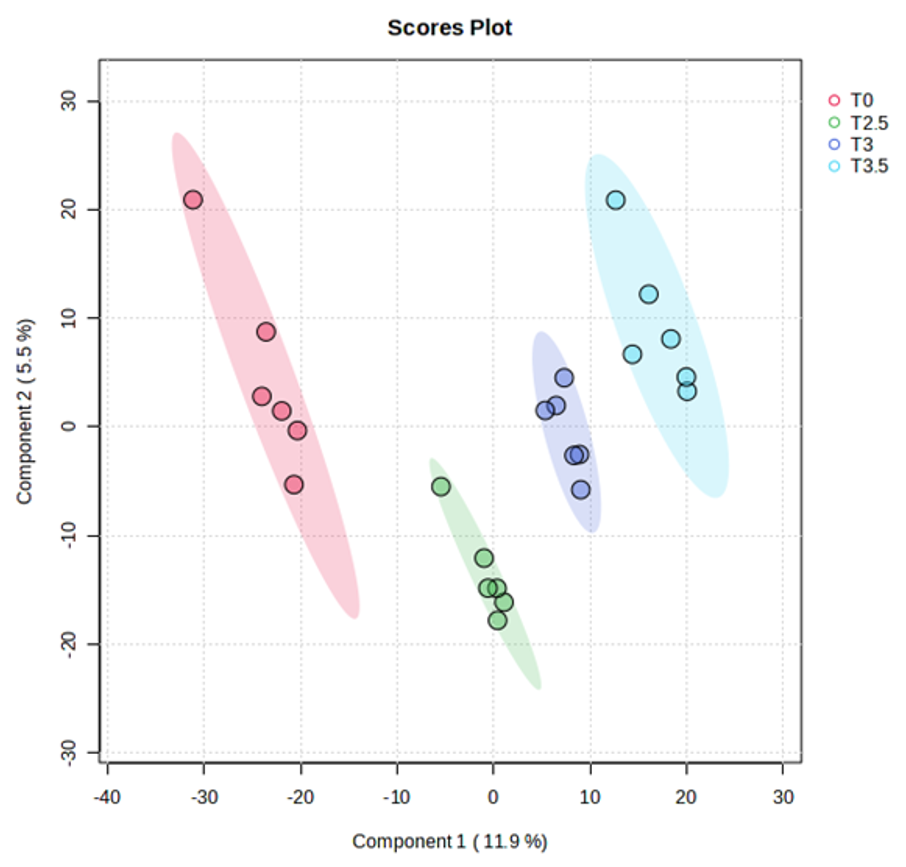



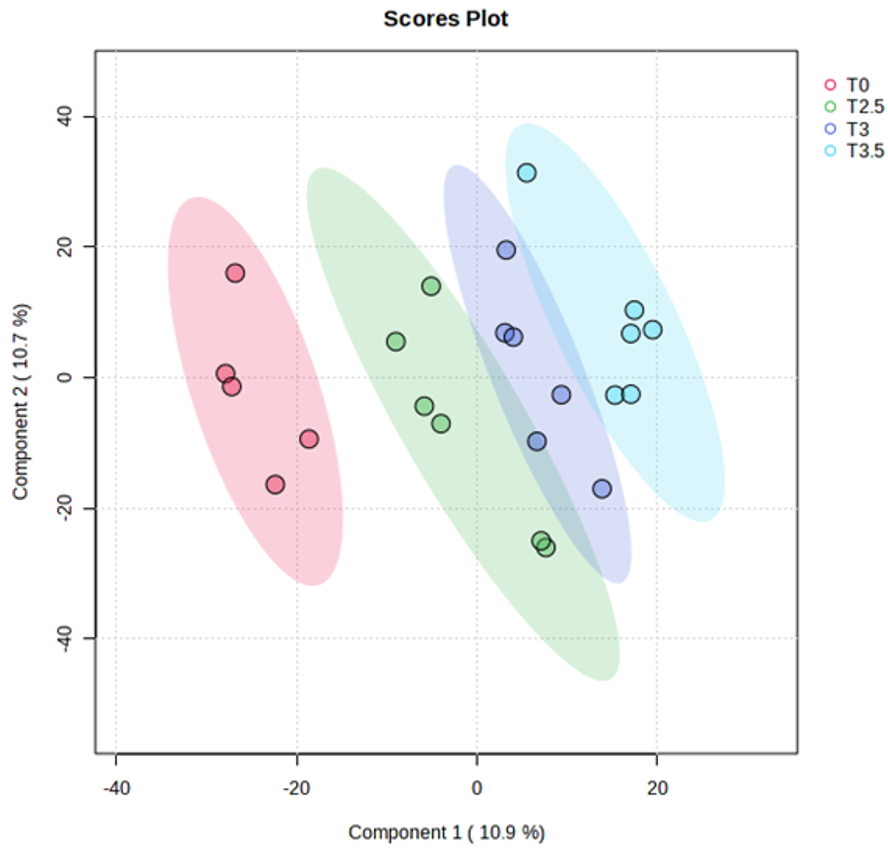


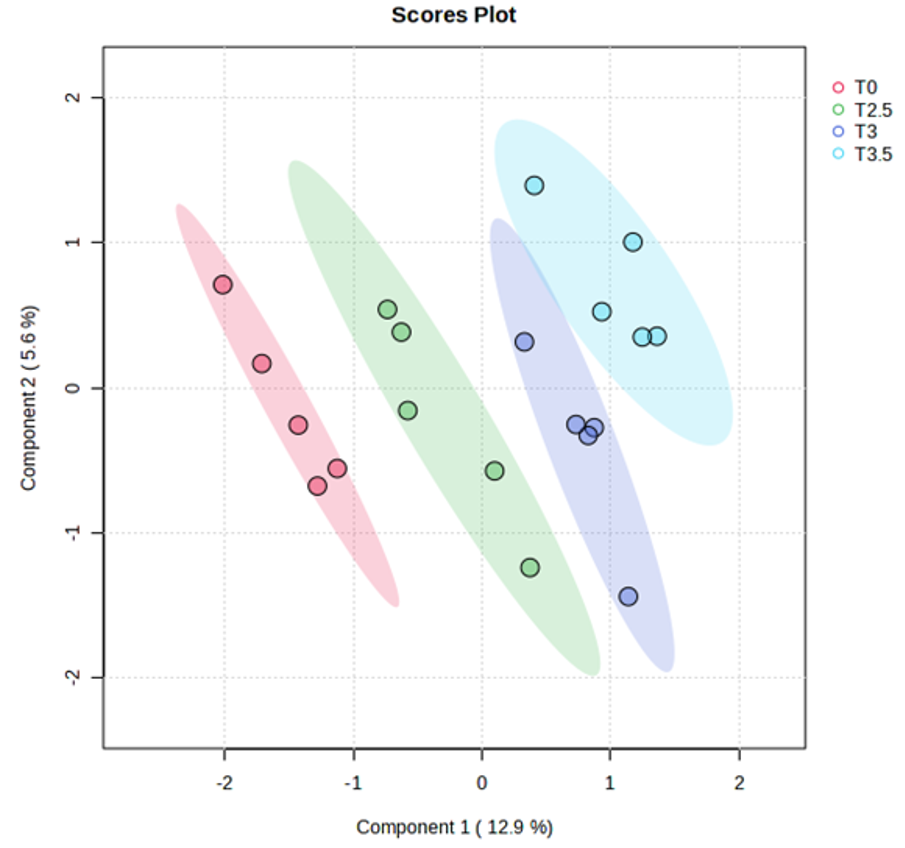



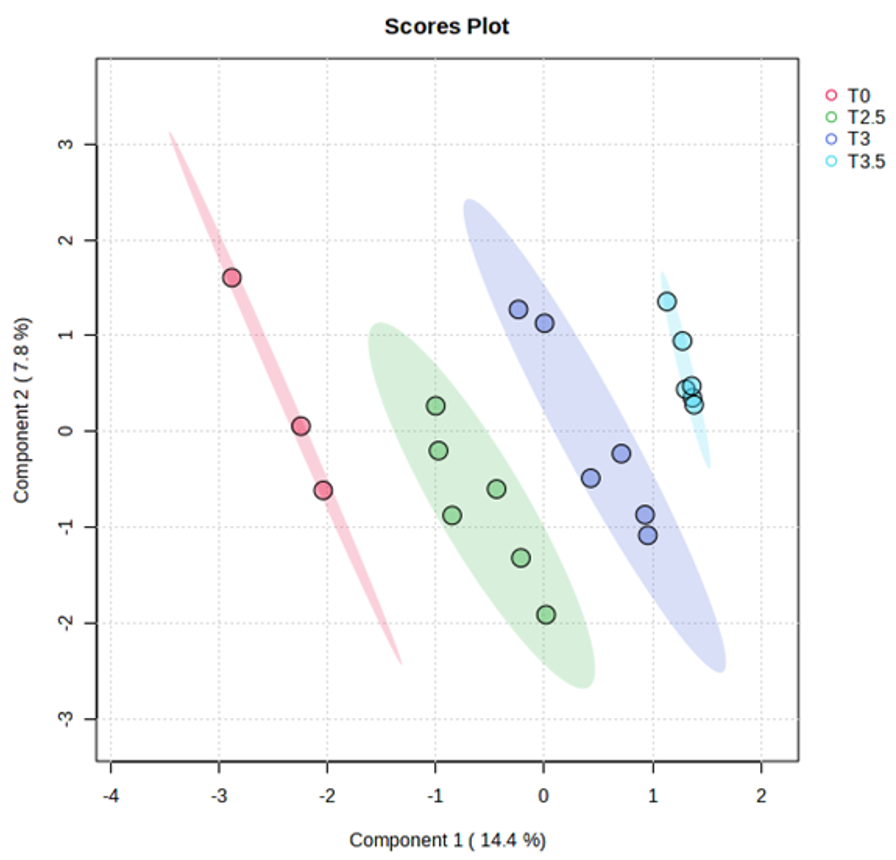


Figure S4. Partial Least Squares-Discriminant Analysis (PLS-DA) of Pharmacometabolomic Data for Plasma Samples from Subjects Administered 1000 mg and 2000 mg Andrographis paniculata Capsules. (a) PLS-DA for 1000 mg in positive mode, (b) 1000 mg in negative mode (One of the T0 sample was excluded due to LCMS instrument stop working.), (c) 2000 mg in positive mode (One set of subject samples (T0, T2.5, T3 and T3.5) were excluded due to sample preparation errors.), and (d) 2000 mg in negative mode (3 samples T0 were excluded due to sample preparation errors), showing clustering trends at pre-dose (T0) and post-dose (T2.5, T3, T3.5) time points



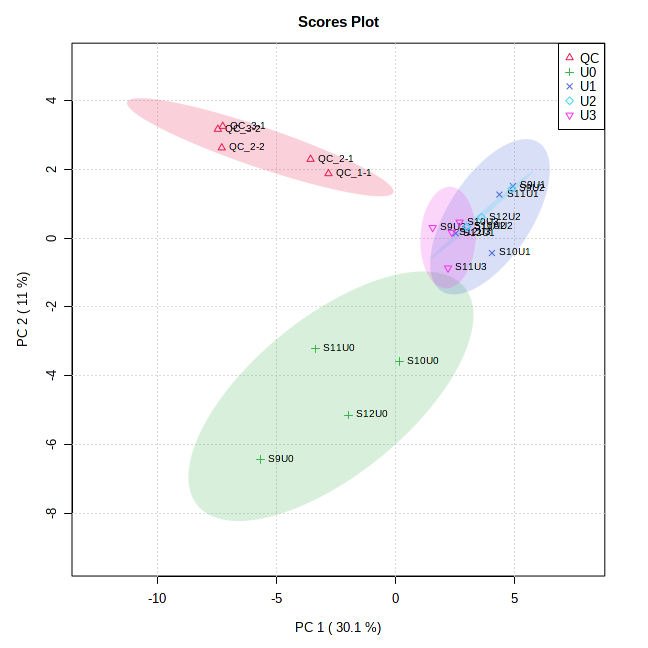


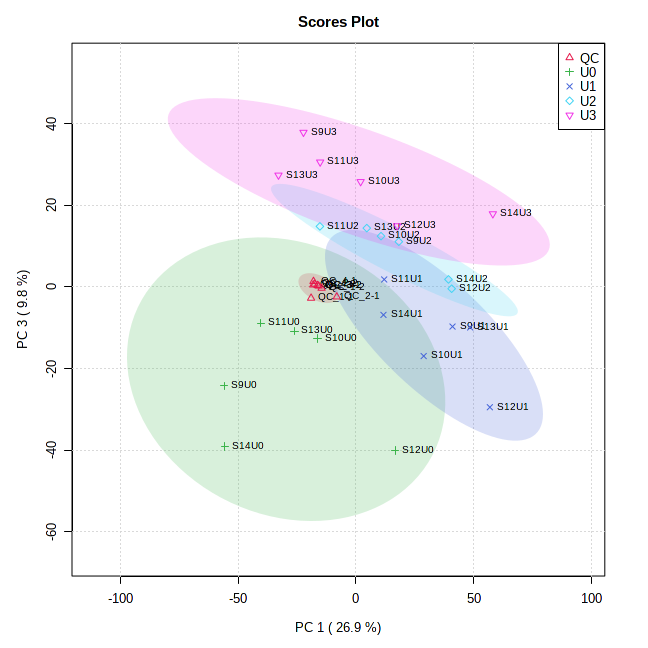


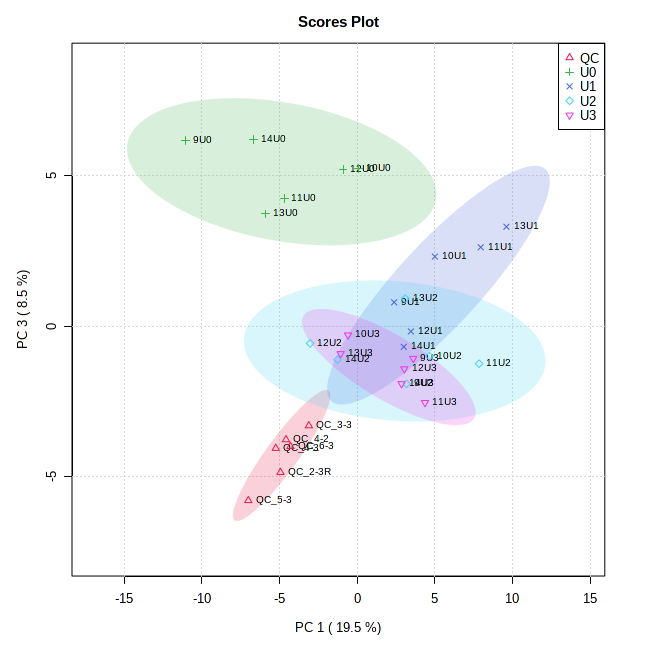


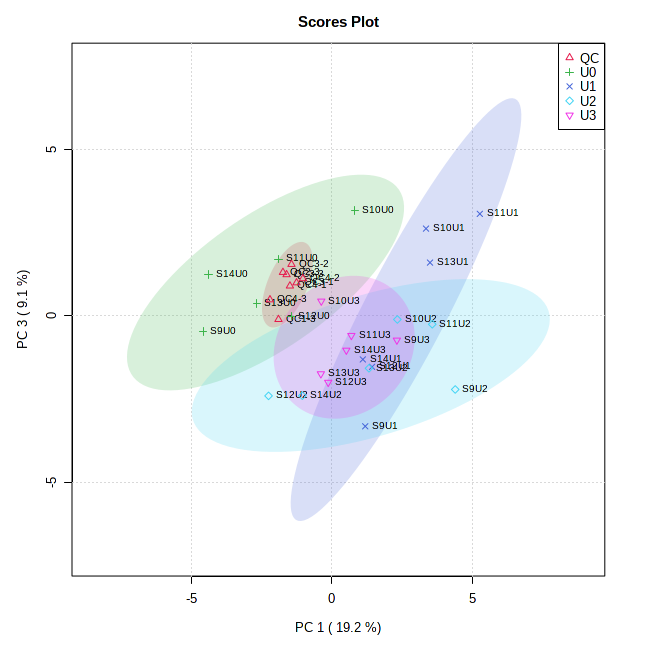


Figure S5. Principal Component Analysis (PCA) of Pharmacometabolomic Data for Urine Samples from Subjects Administered 1000 mg and 2000 mg Andrographis paniculata Capsules. (a) PCA for 1000 mg in positive mode (Two set of subjects’ samples were excluded due to LCMS instrument restart), (b) 1000 mg in negative mode, (c) 2000 mg in positive mode, and (d) 2000 mg in negative mode, showing metabolite distribution at pre-dose (U0) and post-dose (0–4 hr [U1], 4–8 hr [U2], 8–12 hr [U3]) intervals

| a | Batch AP 2000mg plasma positive mode  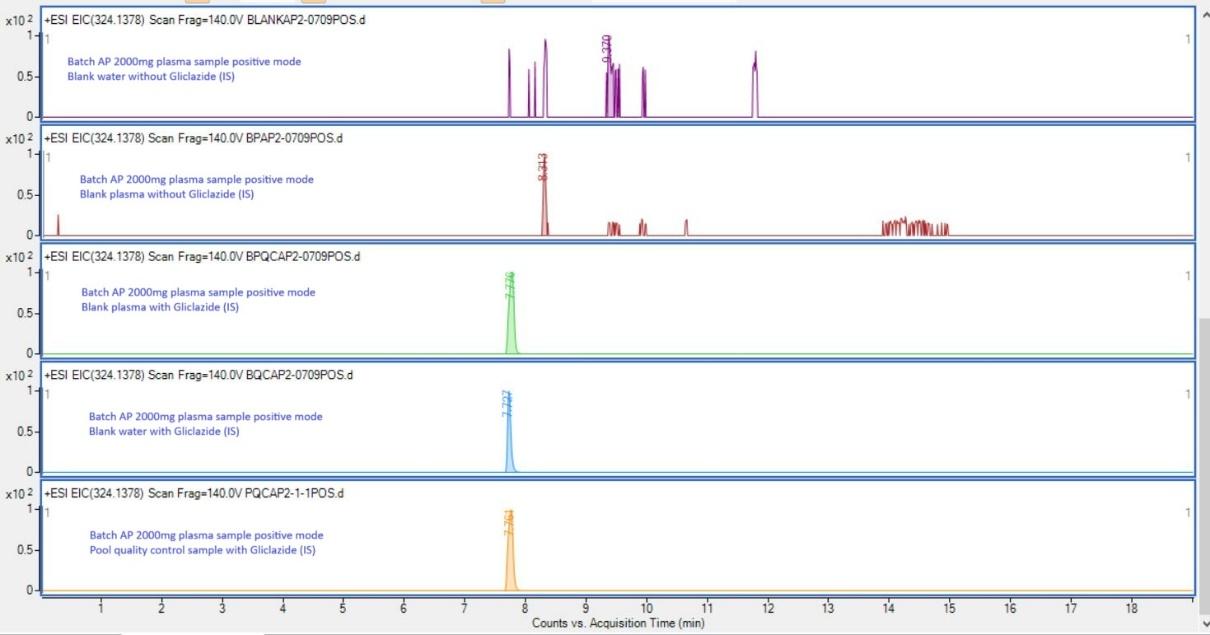  Acceptance criteria: The EIC shown only samples added Gliclazide as internal standard (IS) has peak at m/z 324.1378 at 7.7 min. Accepted this batch for metabolomic data processing. |
| --- | --- |
| b | Batch AP 2000mg plasma negative mode  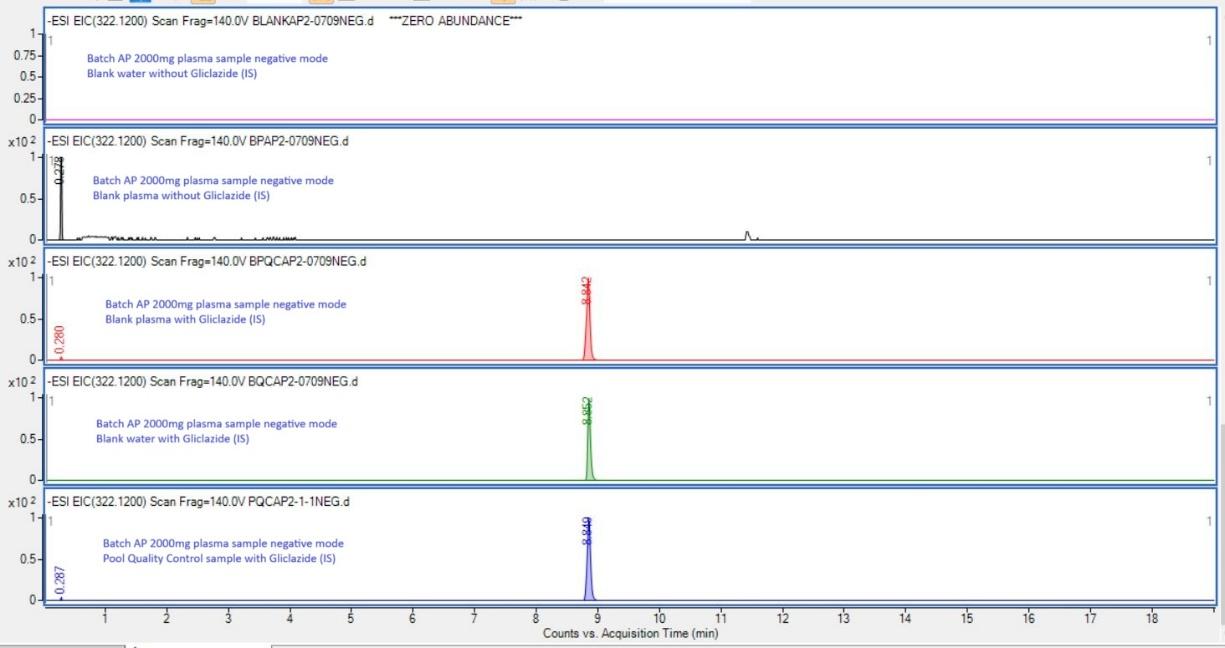  Acceptance criteria: The EIC shown only samples added Gliclazide as internal standard (IS) has peak at m/z 322.12 at 7.7 min. Accepted this batch for metabolomic data processing. |
| C | Batch AP 1000mg plasma positive mode  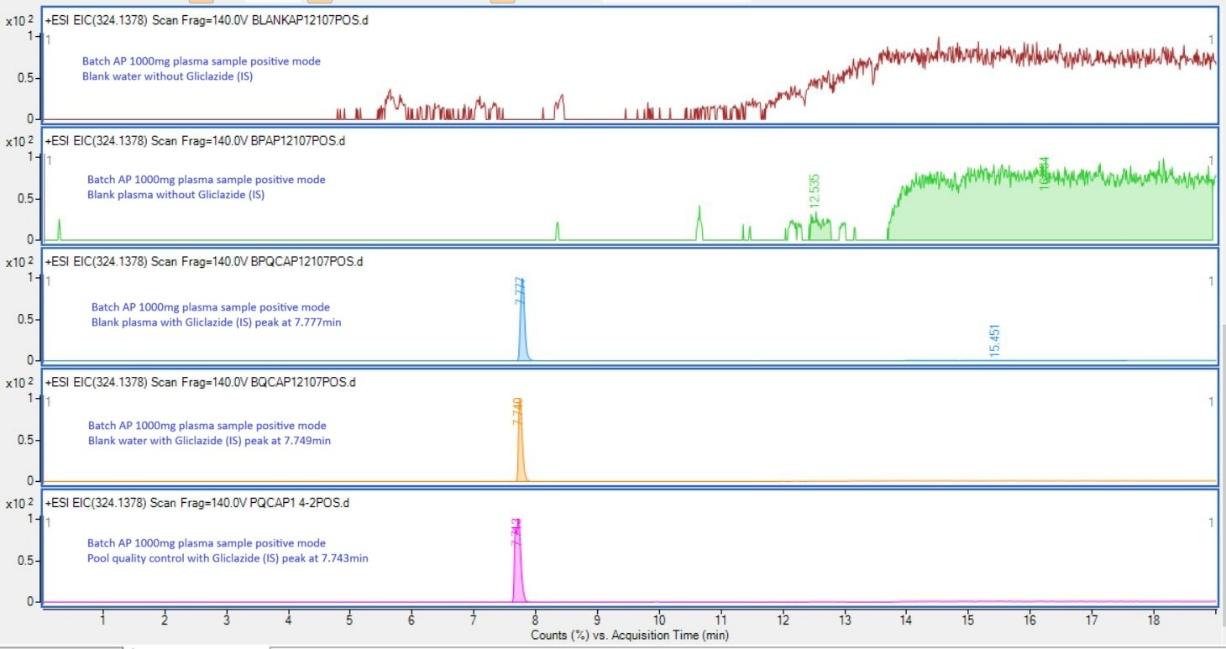Acceptance criteria: The EIC shown only samples added Gliclazide as internal standard (IS) has peak at m/z 324.1378 at 7.7 min. Accepted this batch for metabolomic data processing. |
| D | Batch AP 1000mg plasma positive mode  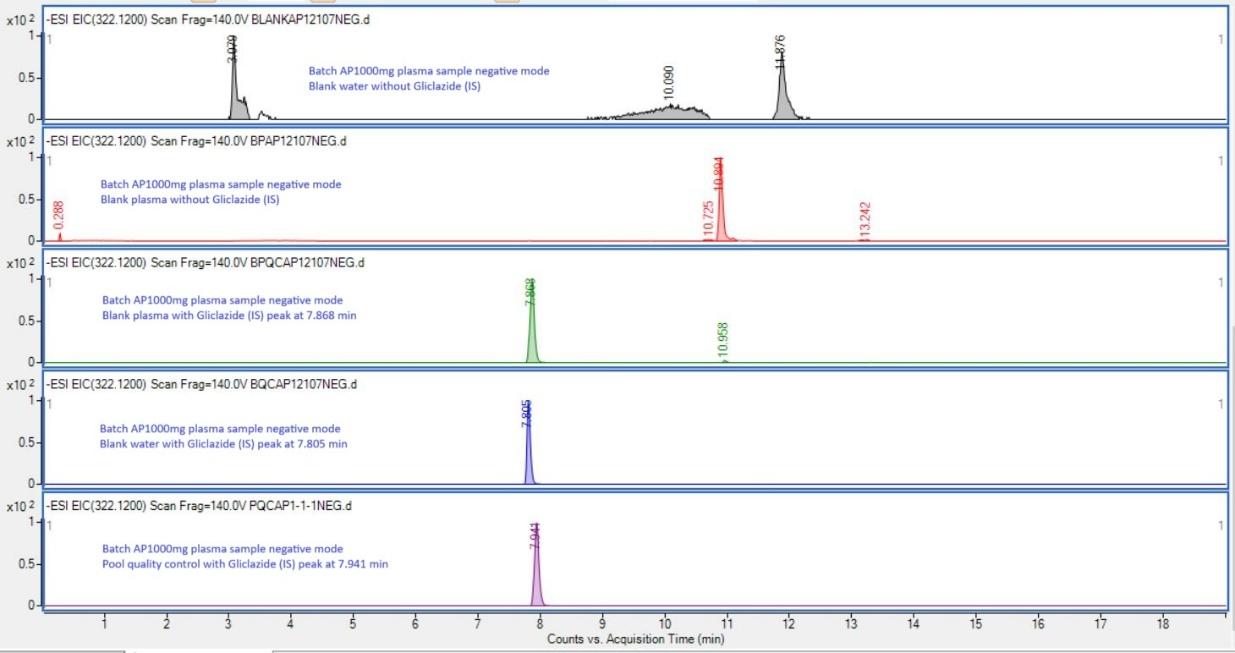Acceptance criteria: The EIC shown only samples added Gliclazide as internal standard (IS) has peak at m/z 322.12 at 7.7 min. Accepted this batch for metabolomic data processing. |
| E | Batch AP 2000mg urine sample positive mode  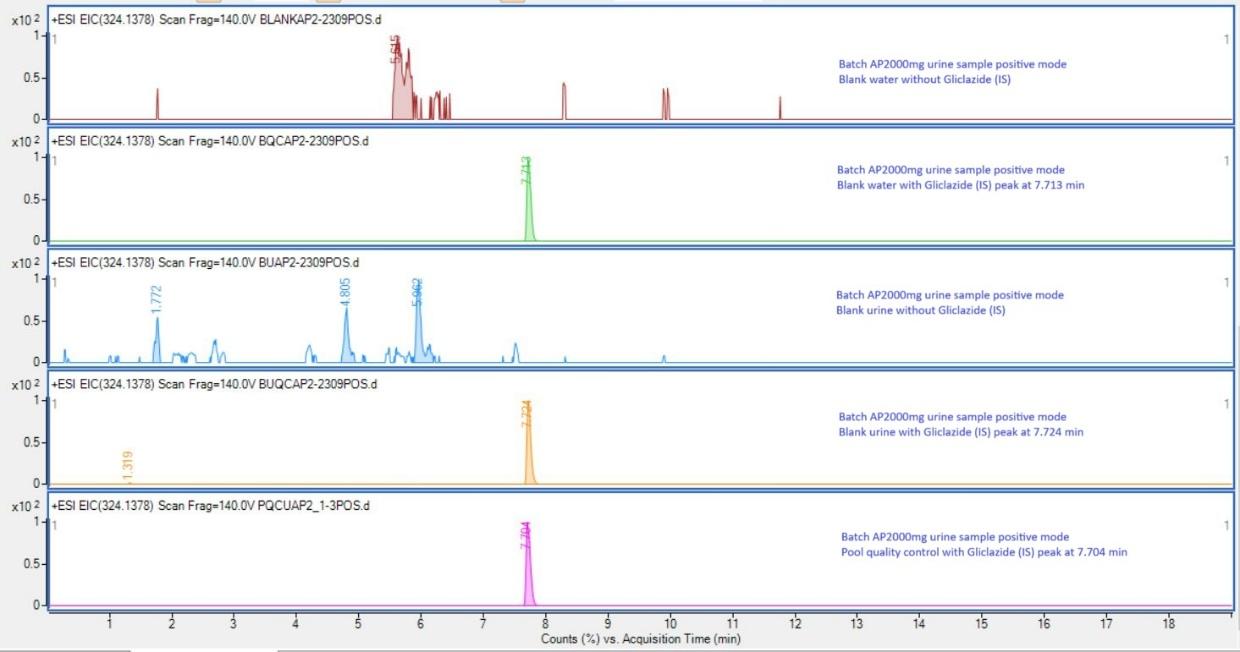Acceptance criteria: The EIC shown only samples added Gliclazide as internal standard (IS) has peak at m/z 324.1378 at 7.7 min. Accepted this batch for metabolomic data processing. |
| F | Batch AP 2000mg urine sample negative mode  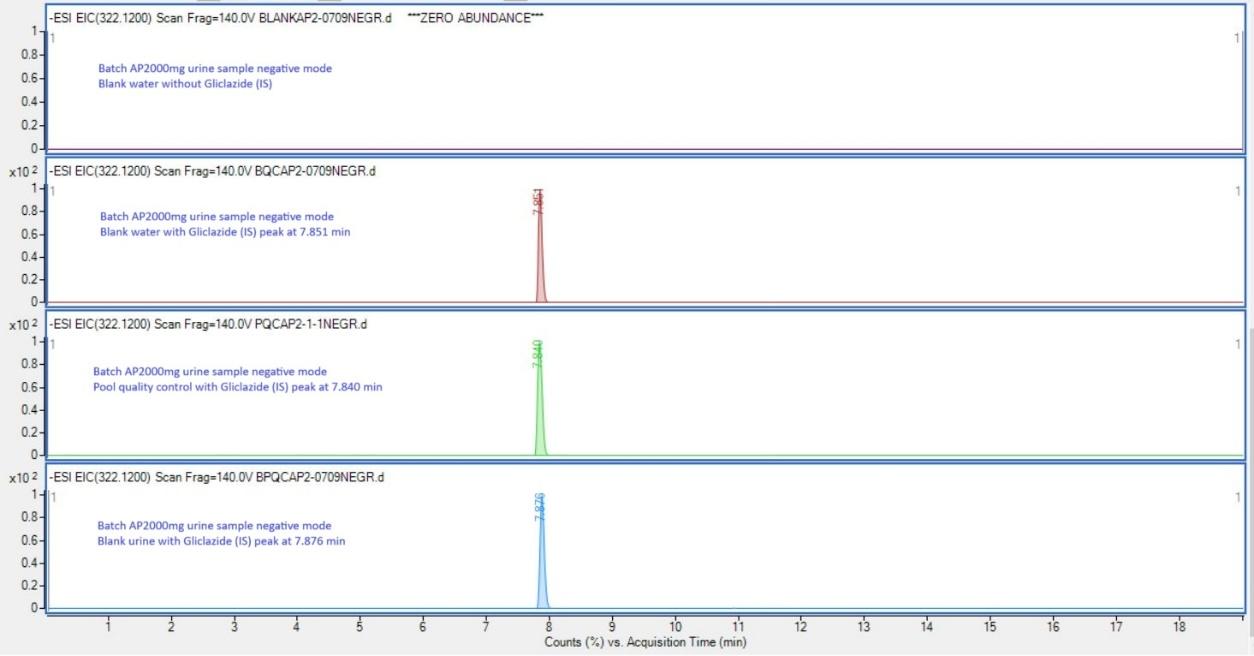Acceptance criteria: The EIC shown only samples added Gliclazide as internal standard (IS) has peak at m/z 322.12 at 7.7 min. Accepted this batch for metabolomic data processing. |
| G | Batch AP 1000mg urine sample positive mode  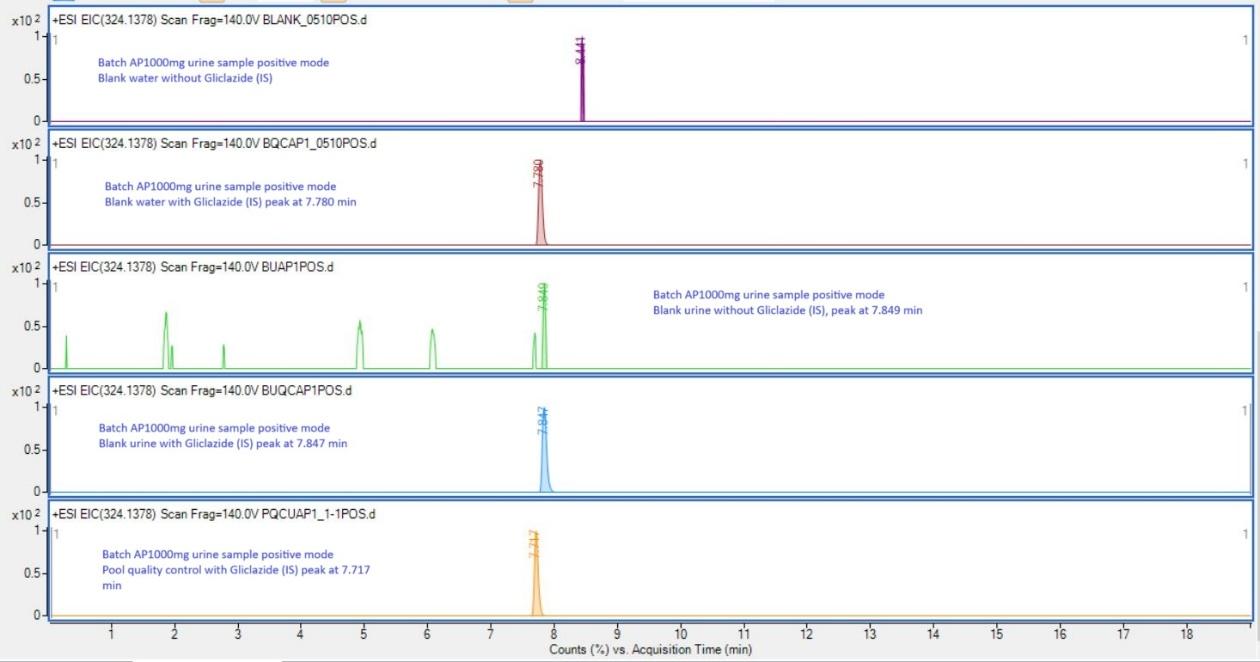Acceptance criteria: The EIC shown only samples added Gliclazide as internal standard (IS) has peak at m/z 324.1378 at 7.7 min. Accepted this batch for metabolomic data processing. |
| H | Batch AP 1000mg urine sample negative mode  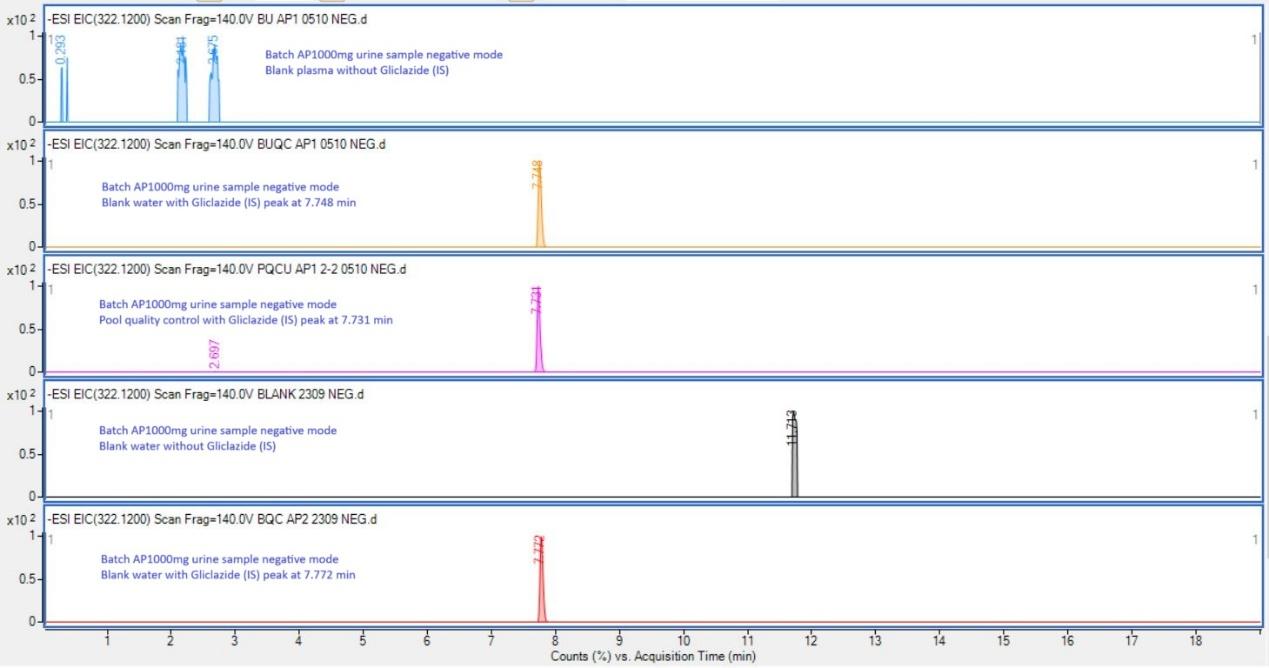Acceptance criteria: The EIC shown only samples added Gliclazide as internal standard (IS) has peak at m/z 322.12 at 7.7 min. Accepted this batch for metabolomic data processing. |

Figure S6: Extracted ion chromatogram (EIC) for blank water and blank plasma with or without Gliclazide internal standard in positive mode and negative mode for eight batches were within the acceptance criteria (a) Batch AP2000mg plasma samples in positive mode, (b) Batch AP2000mg plasma samples in negative mode, (c) Batch AP1000mg plasma samples in positive mode, (d) Batch AP2000mg plasma samples in negative mode, (e) Batch AP 2000mg urine samples in positive mode, (f) Batch AP 2000mg urine samples in negative mode, (g) Batch AP1000mg urine samples in positive mode, (h) Batch AP 1000mg urine samples in negative mode

| A | Batch AP 2000mg plasma positive mode  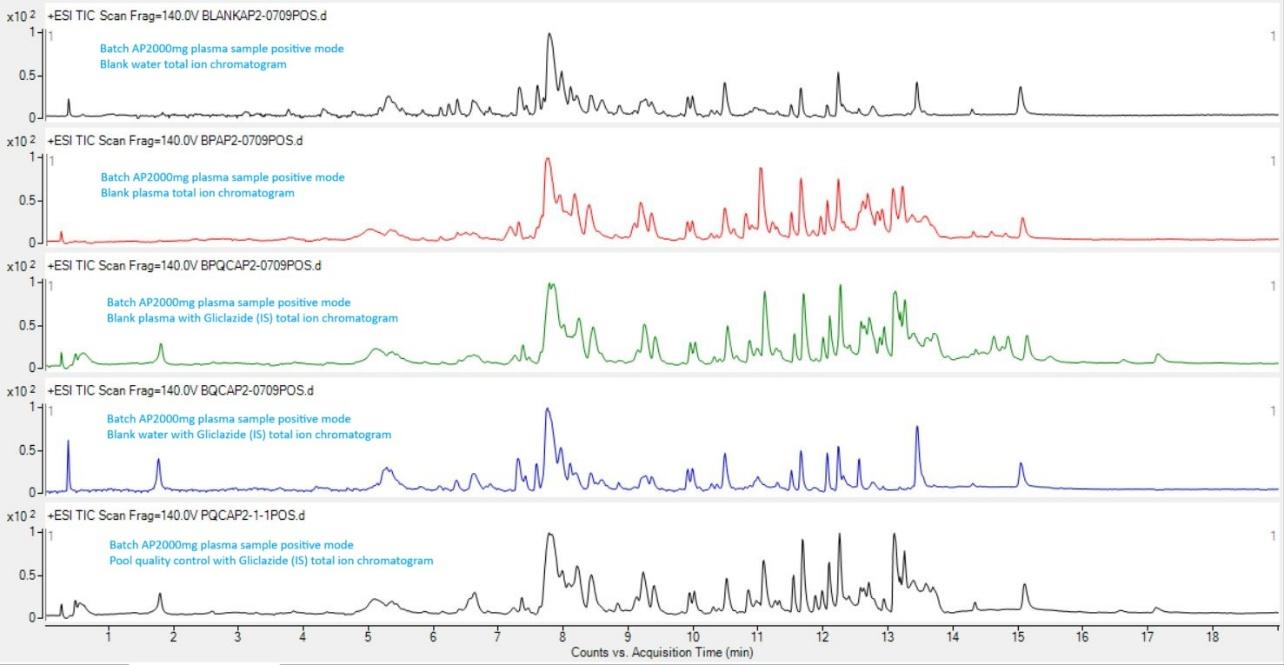  Acceptance criteria: visual inspection accepted |
| --- | --- |
| B | Batch AP 2000mg plasma negative mode  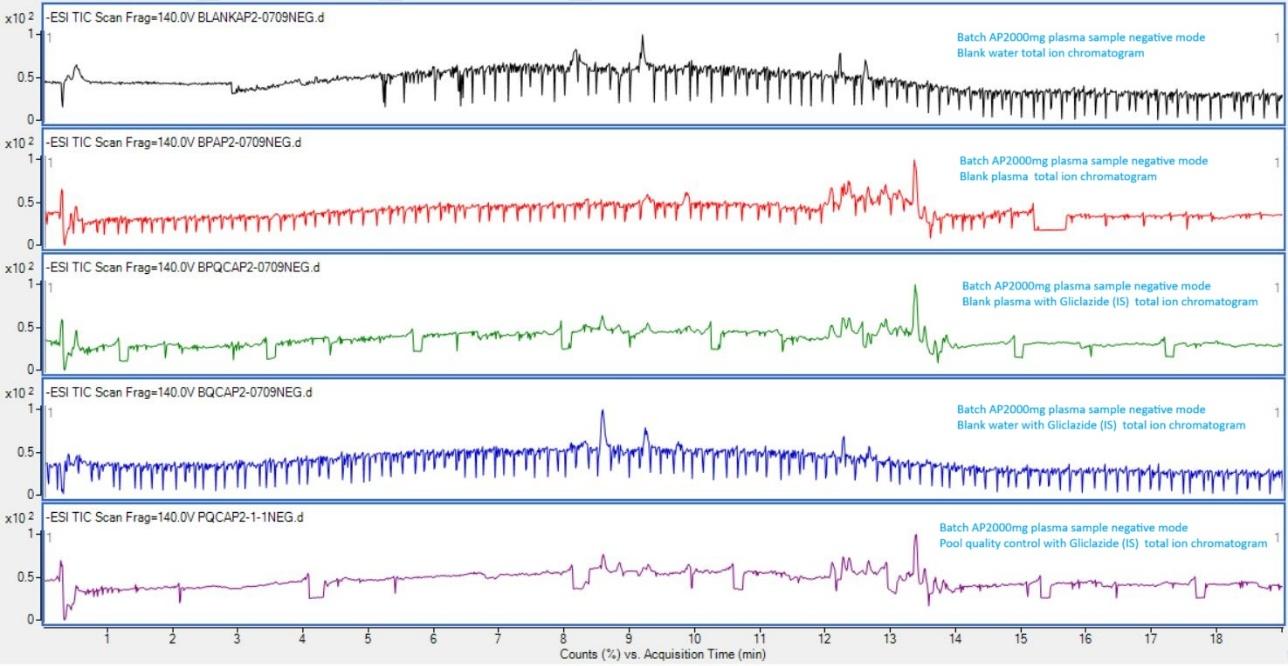  Acceptance criteria: visual inspection accepted |
| C | Batch AP 1000mg plasma positive mode  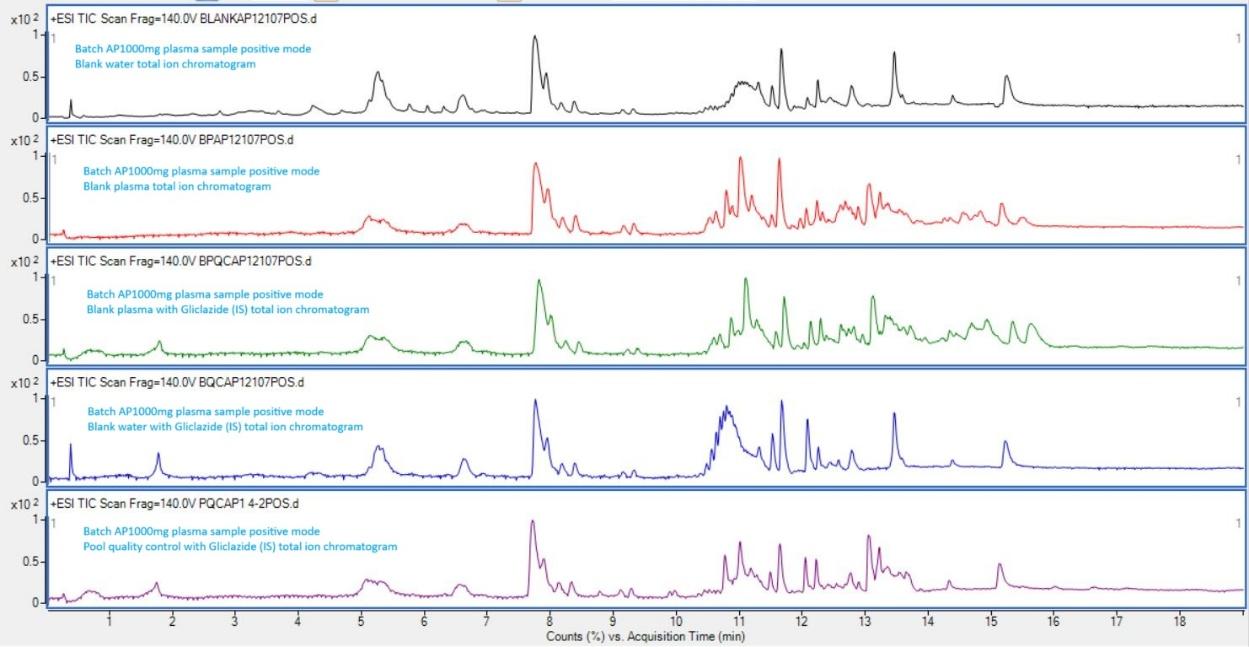Acceptance criteria: visual inspection accepted |
| D | Batch AP 1000mg plasma positive mode  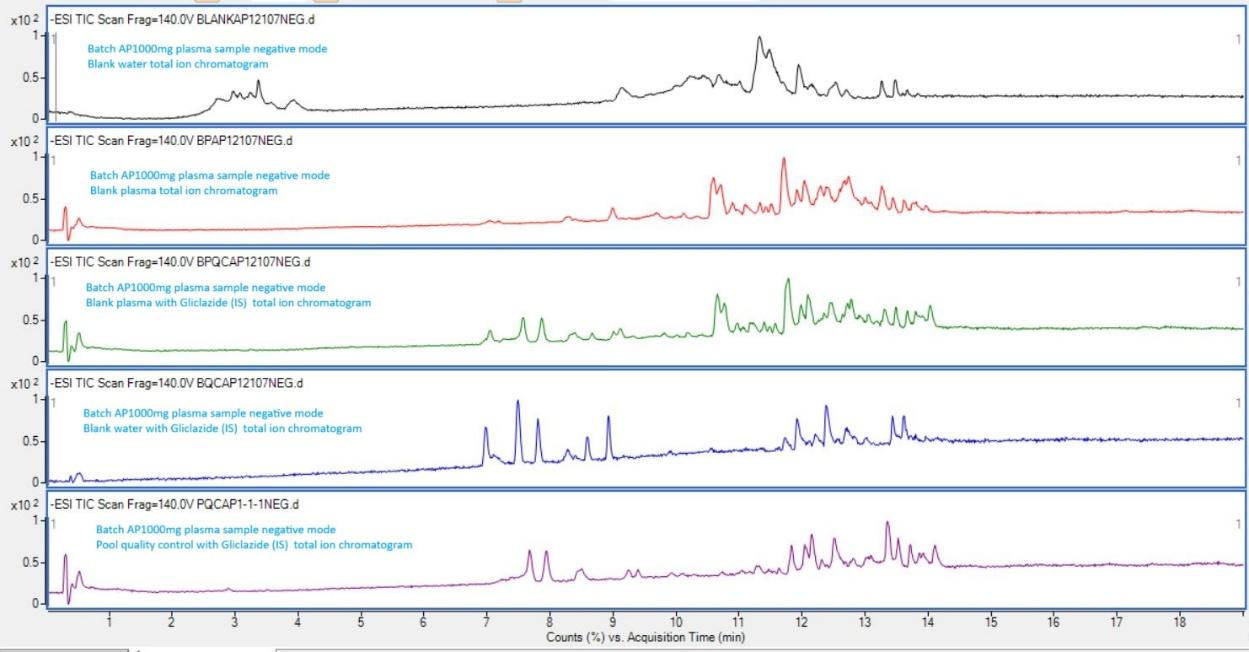Acceptance criteria: visual inspection accepted |
| E | Batch AP 2000mg urine sample positive mode  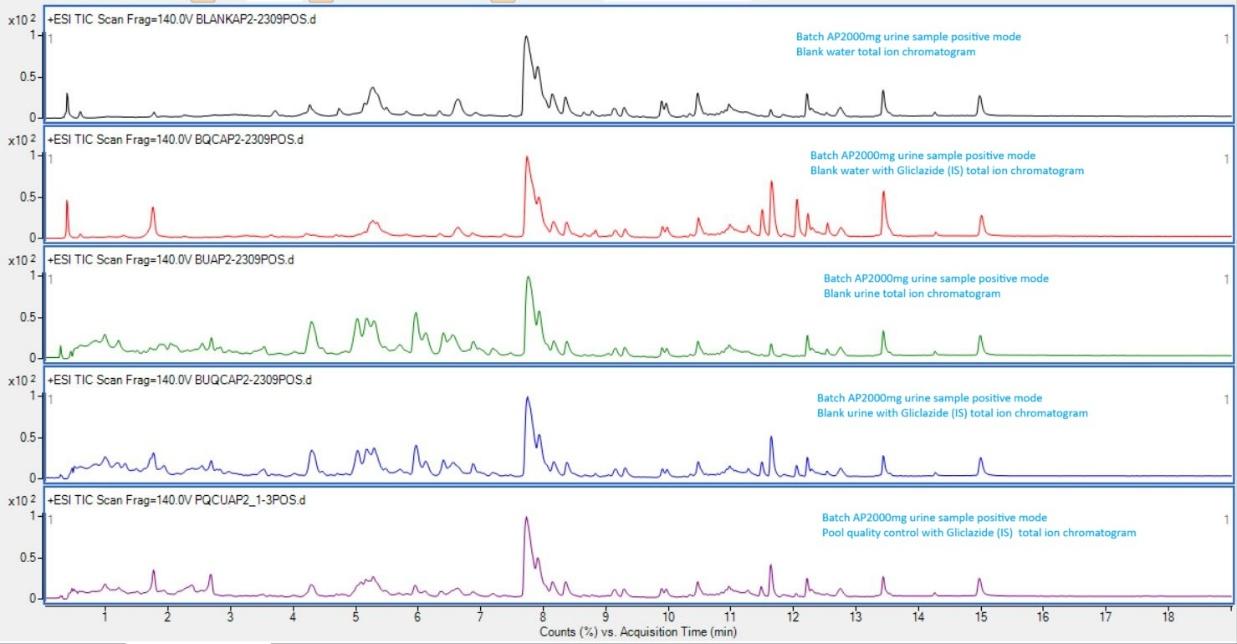  Acceptance criteria: visual inspection accepted |
| F | Batch AP 2000mg urine sample negative mode  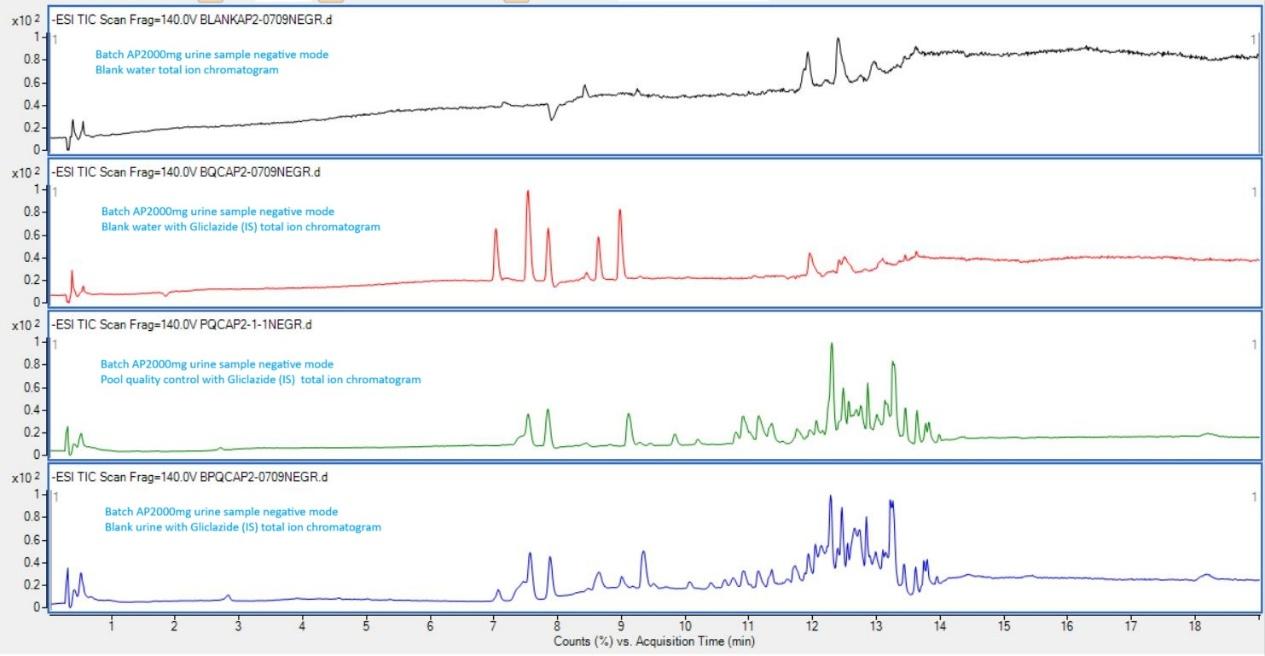Acceptance criteria: visual inspection accepted |
| G | Batch AP 1000mg urine sample positive mode  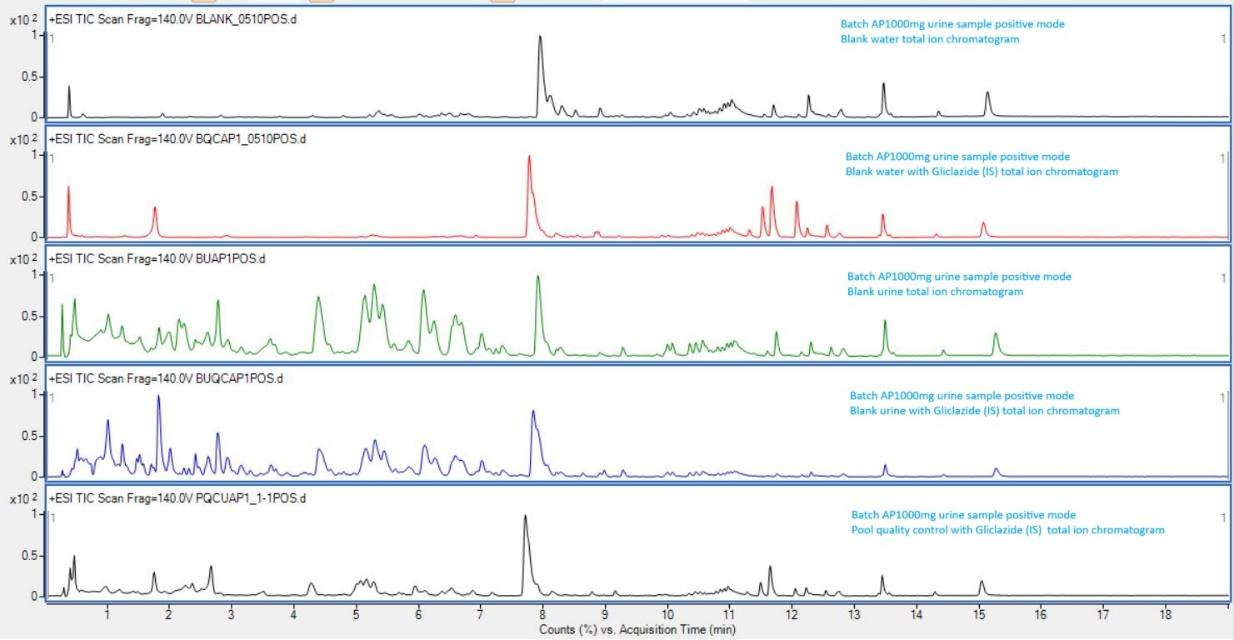Acceptance criteria: visual inspection accepted |
| H | Batch AP 1000mg urine sample negative mode  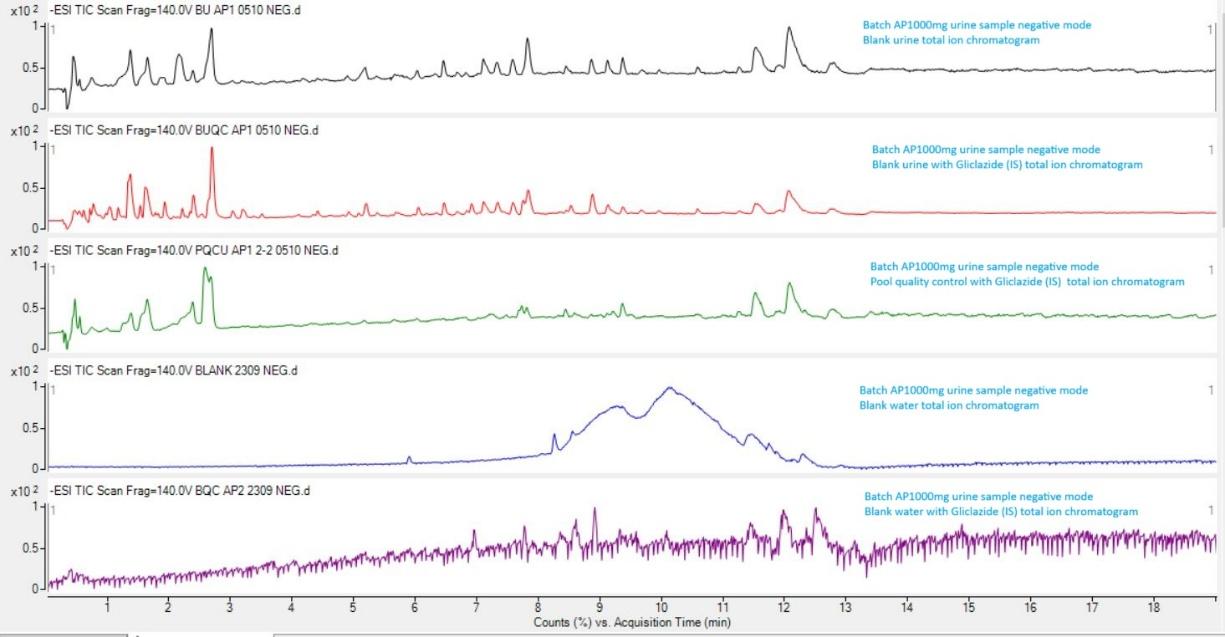  Acceptance criteria: visual inspection accepted |

Figure S7 Total ion chromatogram (TIC) for blank water and blank plasma with or without Gliclazide internal standard in positive mode and negative mode for eight batches were within the acceptance criteria (a) Batch AP2000mg plasma samples in positive mode, (b) Batch AP2000mg plasma samples in negative mode, (c) Batch AP1000mg plasma samples in positive mode, (d) Batch AP2000mg plasma samples in negative mode, (e) Batch AP 2000mg urine samples in positive mode, (f) Batch AP 2000mg urine samples in negative mode, (g) Batch AP1000mg urine samples in positive mode, (h) Batch AP 1000mg urine samples in negative mode

|  | Overlay pool quality Control sample | Overlay pool quality control samples and subject samples |
| --- | --- | --- |
| A | Batch AP 2000mg plasma positive mode  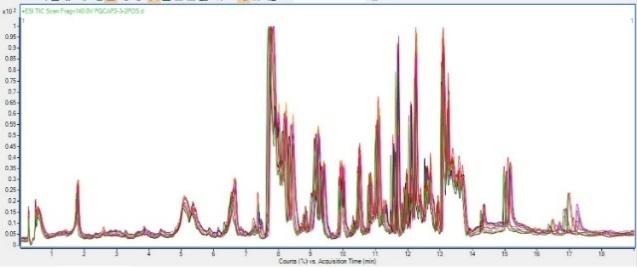  Acceptance criteria: visual inspection for variability  Accepted for metabolomics analysis | 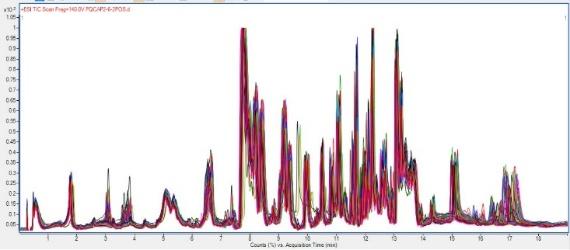  Acceptance criteria: visual inspection for variability  Accepted for metabolomics analysis |
| B | Batch AP 2000mg plasma negative mode  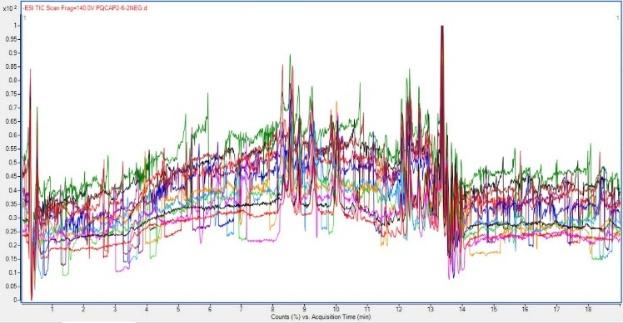  Acceptance criteria: visual inspection for variability  Accepted for metabolomics analysis | 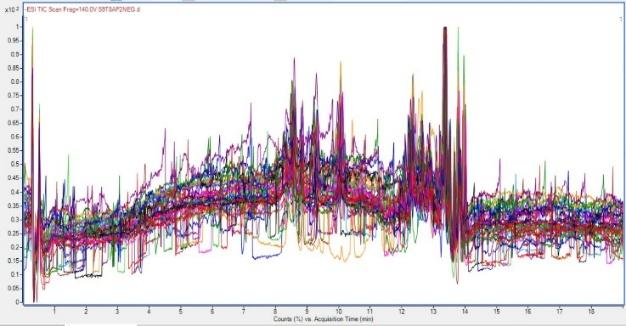  Acceptance criteria: visual inspection for variability  Accepted for metabolomics analysis |
| C | Batch AP 1000mg plasma positive mode  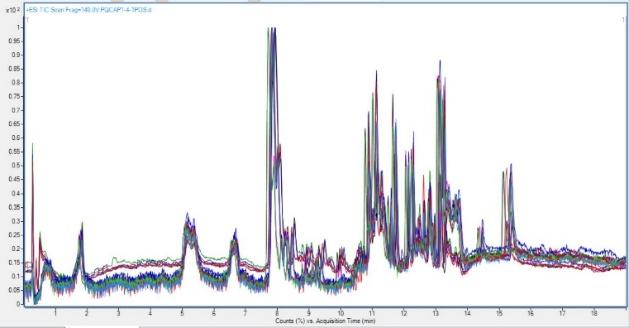  Acceptance criteria: visual inspection for variability  Accepted for metabolomics analysis | 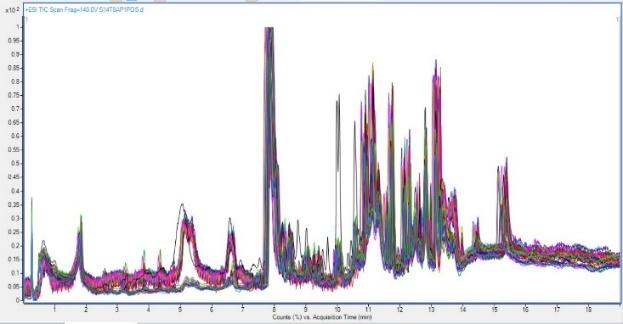  Acceptance criteria: visual inspection for variability  Accepted for metabolomics analysis |
| D | Batch AP 1000mg plasma negative mode  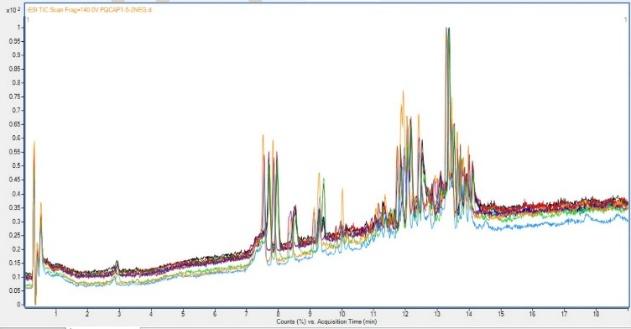  Acceptance criteria: visual inspection for variability  Accepted for metabolomics analysis | 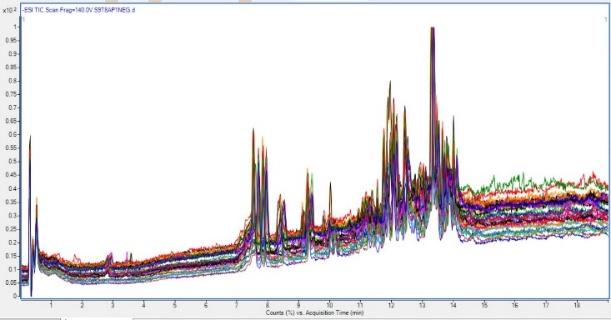  Acceptance criteria: visual inspection for variability  Accepted for metabolomics analysis |
| E | Batch AP 2000mg urine sample positive mode  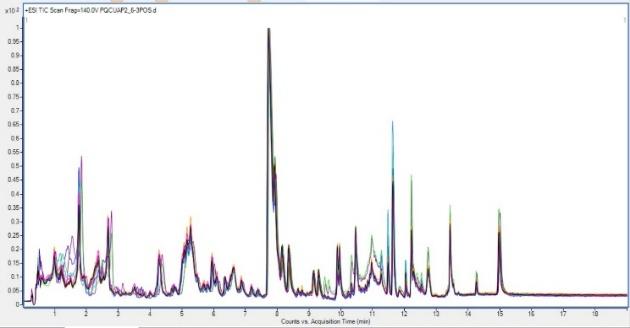  Acceptance criteria: visual inspection for variability  Accepted for metabolomics analysis | 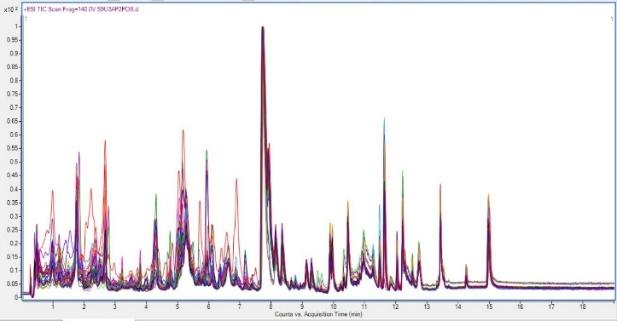  Acceptance criteria: visual inspection for variability  Accepted for metabolomics analysis |
| F | Batch AP 2000mg urine sample negative mode  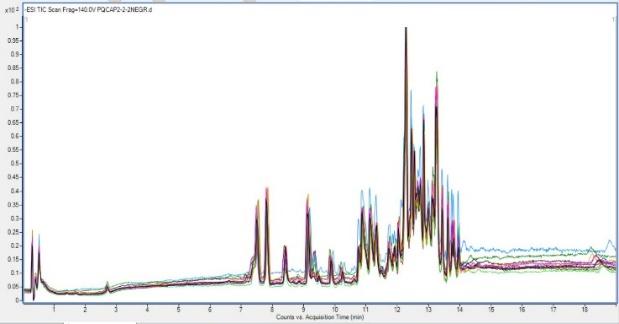  Acceptance criteria: visual inspection for variability  Accepted for metabolomics analysis | 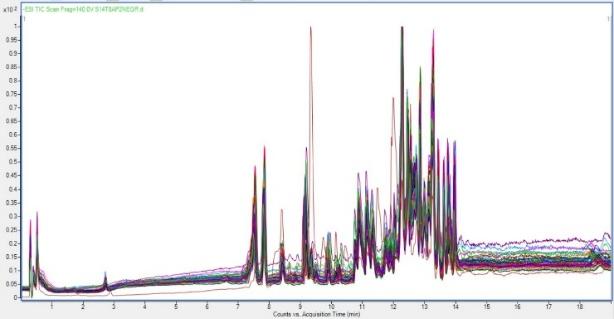  Acceptance criteria: visual inspection for variability  Accepted for metabolomics analysis |
| G | Batch AP 1000mg urine sample positive mode  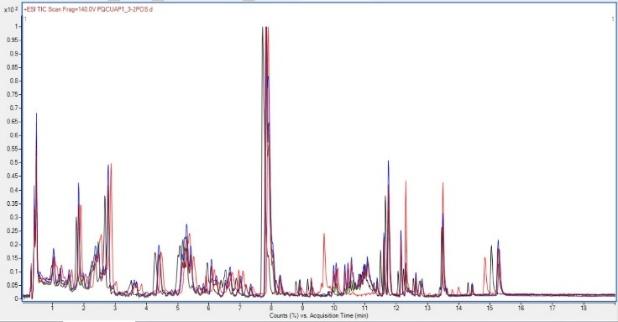  Acceptance criteria: visual inspection for variability  Accepted for metabolomics analysis | 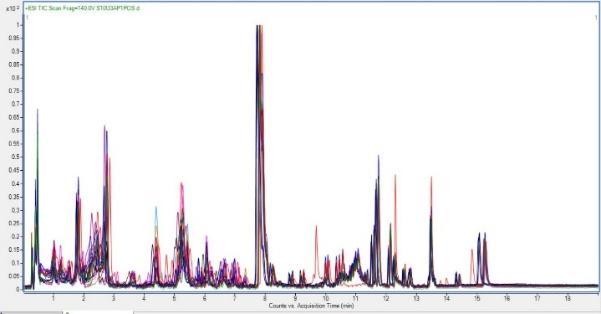  Acceptance criteria: visual inspection for variability  Accepted for metabolomics analysis |
| H | Batch AP 1000mg urine sample negative mode  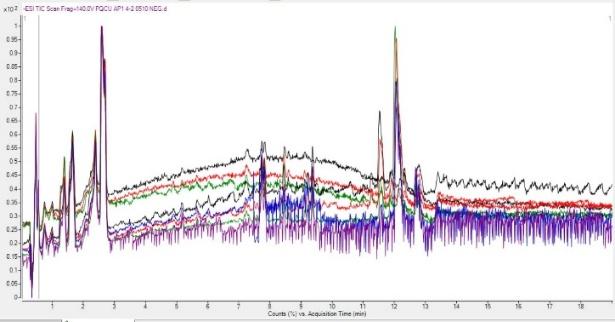  Acceptance criteria: visual inspection for variability  Peaks variation found, may explore metabolomics analysis | 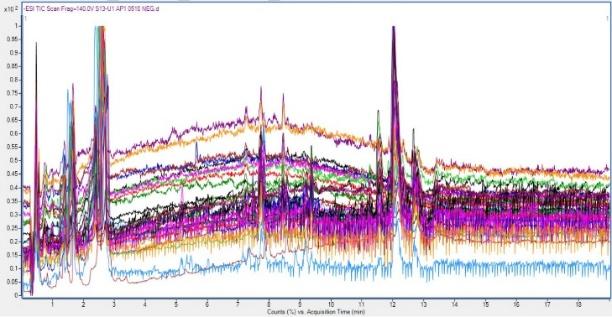  Acceptance criteria: visual inspection for variability  Peaks variation found, may explore metabolomics analysis |

Figure S8: Overlay of pool quality control samples (PQC) and PQC plus subject samples for eight batches were within the acceptance criteria (a) Batch AP2000mg plasma samples in positive mode, (b) Batch AP2000mg plasma samples in negative mode, (c) Batch AP1000mg plasma samples in positive mode, (d) Batch AP2000mg plasma samples in negative mode, (e) Batch AP 2000mg urine samples in positive mode, (f) Batch AP 2000mg urine samples in negative mode, (g) Batch AP1000mg urine samples in positive mode, (h) Batch AP 1000mg urine samples in negative mode

Table S1. Total number of samples for AP capsule quantitative analysis, pharmacokinetics analysis and pharmacometabolomic analysis

| **Analysis** | **Capsules** | **Sample types** | **Biological samples** | **Number/ time points** | **Number of LCMS analysis** |
| --- | --- | --- | --- | --- | --- |
| Biomarkers quantification |  | Capsules solution | 5 brands | 3 | 15 |
| Pharmacokinetics | AP2000mg | Plasma | 5 subjects | 15 time points | 75 |
| Pharmacometabolomics | AP2000mg | Plasma | 6 subjects | 4 time points | 24 positive mode |
|  |  | Plasma | 6 subjects | 4 time points | 24 negative mode |
|  | AP1000mg | Plasma | 6 subjects | 4 time points | 24 positive mode |
|  |  | Plasma | 6 subjects | 4 time points | 24 negative mode |
|  | AP2000mg | Urine | 6 subjects | 4 time points | 24 positive mode |
|  |  | Urine | 6 subjects | 4 time points | 24 negative mode |
|  | AP1000mg | Urine | 6 subjects | 4 time points | 24 positive mode |
|  |  | Urine | 6 subjects | 4 time points | 24 negative mode |
| **TOTAL SAMPLE RUN** |  |  |  |  | **282 runs** |

Table S2. Calibration curve and quality control sample concentrations for Andrographis paniculata capsule bioactive compounds.

| **Andrographolide** | | | | | |
| --- | --- | --- | --- | --- | --- |
| **Sample Name** | **Sample Type** | **Response** | **Final Concentration** | **Exp Concentration** | **Accuracy** |
| CC1 | Calibration | 77725 | 59.30 | 62.81 | 94.41 |
| CC2 | Calibration | 92666 | 73.50 | 87.94 | 83.58 |
| CC3 | Calibration | 261235 | 260.53 | 276.38 | 94.27 |
| CC4 | Calibration | 507867 | 586.63 | 628.13 | 93.39 |
| CC5 | Calibration | 1783301 | 2718.78 | 2512.50 | 108.21 |
| CC6 | Calibration | 4045501 | 7391.45 | 7537.50 | 98.06 |
| CC7 | Calibration | 5122964 | 9861.41 | 10050.00 | 98.12 |
| CC8 | Calibration | 6329665 | 12767.22 | 12562.50 | 101.63 |
| LQC | QC | 2109187 | 3337.11 | 3266.25 | 102.17 |
| HQC | QC | 3568434 | 6341.52 | 6532.50 | 97.08 |

| **Neoandrographolide** | | | | | |
| --- | --- | --- | --- | --- | --- |
|  | **Sample Type** | **Response** | **Final Conc** | **Exp Conc** | **Accuracy** |
| CC1 | Calibration | 77725 | 59.30 | 62.81 | 94.41 |
| CC2 | Calibration | 92666 | 73.50 | 87.94 | 83.58 |
| CC3 | Calibration | 261235 | 260.53 | 276.38 | 94.27 |
| CC4 | Calibration | 507867 | 586.63 | 628.13 | 93.39 |
| CC5 | Calibration | 1783301 | 2718.78 | 2512.50 | 108.21 |
| CC6 | Calibration | 4045501 | 7391.45 | 7537.50 | 98.06 |
| CC7 | Calibration | 5122964 | 9861.41 | 10050.00 | 98.12 |
| CC8 | Calibration | 6329665 | 12767.22 | 12562.50 | 101.63 |
| LQC | QC | 2109187 | 3337.11 | 3266.25 | 102.17 |
| HQC | QC | 3568434 | 6341.52 | 6532.50 | 97.08 |

| **14-deoxyandrographolide** | | | | | |
| --- | --- | --- | --- | --- | --- |
| **Sample Name** | **Sample Type** | **Response** | **Final Conc** | **Exp Conc** | **Accuracy** |
| CC1 | Calibration | 13455 | 66.77 | 61.88 | 107.91 |
| CC2 | Calibration | 27521 | 134.16 | 123.75 | 108.41 |
| CC3 | Calibration | 64182 | 311.20 | 309.38 | 100.59 |
| CC4 | Calibration | 122525 | 597.32 | 618.75 | 96.54 |
| CC5 | Calibration | 676335 | 3655.07 | 3712.50 | 98.45 |
| CC6 | Calibration | 895843 | 5115.34 | 4950.00 | 103.34 |
| CC7 | Calibration | 1024432 | 6076.30 | 6187.50 | 98.20 |
| CC8 | Calibration | 1556186 | 12389.78 | 12375.00 | 100.12 |
| LQC | QC | 278087 | 1388.72 | 1485.00 | 93.52 |
| HQC | QC | 394438 | 2011.15 | 2475.00 | 81.26 |

Table S3. Global metabolomics LC-MS analysis sequence table for (i) plasma samples and (ii) urine samples from subjects administered 1000 mg and 2000 mg Andrographis paniculata capsules.

(i)

| **AP 1000mg plasma sample** | | | **Run Acceptance** | | **AP 2000mg plasma sample** | | | **Run Acceptance** | |
| --- | --- | --- | --- | --- | --- | --- | --- | --- | --- |
| **Sample name** | **Time** | **Vial label** | **+ve** | **-ve** | **Sample name** | **Time** | **Vial label** | **+ve** | **-ve** |
| Pool Quality Control |  | AP1PQC1 | √ | √ | Pool Quality Control |  | AP2PQC1 | √ | √ |
| Pool Quality Control |  | AP1PQC2 | √ | √ | Pool Quality Control |  | AP2PQC2 | √ | √ |
| Pool Quality Control |  | AP1PQC3 | √ | √ | Pool Quality Control |  | AP2PQC3 | √ | √ |
| Pool Quality Control |  | AP1PQC4 | √ | √ | Pool Quality Control |  | AP2PQC4 | √ | √ |
| Pool Quality Control |  | AP1PQC5 | √ | √ | Pool Quality Control |  | AP2PQC5 | √ | √ |
| Subject 4 | 2.5 | AP1S4T2.5 | √ | √ | Subject 1 | 0 | AP2S1T0 | √ | √ |
| Subject 4 | 0 | AP1S4T0 | √ | √ | Subject 1 | 2.5 | AP2S1T2.5 | √ | √ |
| Subject 4 | 3 | AP1S4T3 | √ | √ | Subject 1 | 3 | AP2S1T3 | √ | √ |
| Subject 2 | 2.5 | AP1S2T2.5 | √ | √ | Subject 1 | 3.5 | AP2S1T3.5 | √ | √ |
| Pool Quality Control |  | AP1PQC6 | √ | √ | Pool Quality Control |  | AP2PQC6 | √ | √ |
| Subject 5 | 3.5 | AP1S5T3.5 | √ | √ | Subject 2 | 3.5 | AP2S2T3.5 | √ | √ |
| Subject 6 | 0 | AP1S6T0 | √ | √ | Subject 2 | 3 | AP2S2T3 | √ | √ |
| Subject 3 | 0 | AP1S3T0 | √ | √ | Subject 2 | 2.5 | AP2S2T2.5 | √ | √ |
| Subject 4 | 3.5 | AP1S4T3.5 | √ | √ | Subject 2 | 0 | AP2S2T0 | √ | √ |
| Pool Quality Control |  | AP1PQC7 | √ | √ | Pool Quality Control |  | AP2PQC7 | √ | √ |
| Subject 1 | 0 | AP1S1T0 | √ | √ | Subject 3 | 0 | AP2S3T0 | √ | √ |
| Subject 2 | 3.5 | AP1S2T3.5 | √ | √ | Subject 3 | 3.5 | AP2S3T3.5 | √ | √ |
| Subject 3 | 2.5 | AP1S3T2.5 | √ | √ | Subject 3 | 3 | AP2S3T3 | √ | √ |
| Subject 1 | 3 | AP1S1T3 | √ | √ | Subject 3 | 2.5 | AP2S3T2.5 | √ | √ |
| Pool Quality Control |  | AP1PQC8 | √ | √ | Pool Quality Control |  | AP2PQC8 | √ | √ |
| Subject 2 | 0 | AP1S2T0 | √ | √ | Subject 5 | 3 | AP2S5T3 | √ | √ |
| Subject 3 | 3 | AP1S3T3 | √ | √ | Subject 4 | 3 | AP2S4T3 | √ | √ |
| Subject 1 | 2.5 | AP1S1T2.5 | √ | √ | Subject 5 | 2.5 | AP2S5T2.5 | √ | √ |
| Subject 5 | 2.5 | AP1S5T2.5 | √ | √ | Subject 4 | 3.5 | AP2S4T3.5 | √ | √ |
| Pool Quality Control |  | AP1PQC9 | √ | √ | Pool Quality Control |  | AP2PQC9 | √ | √ |
| Subject 1 | 3.5 | AP1S1T3.5 | √ | √ | Subject 4 | 2.5 | AP2S4T2.5 | √ | √ |
| Subject 6 | 3.5 | AP1S6T3.5 | √ | √ | Subject 5 | 3.5 | AP2S5T3.5 | √ | √ |
| Subject 6 | 3 | AP1S6T3 | √ | √ | Subject 4 | 0 | AP2S4T0 | √ | X |
| Subject 5 | 3 | AP1S5T3 | √ | √ | Subject 5 | 0 | AP2S5T0 | √ | X |
| Pool Quality Control |  | AP1PQC10 | √ | √ | Pool Quality Control |  | AP2PQC10 | √ | X |
| Subject 2 | 3 | AP1S2T3 | √ | √ | Subject 6 | 0 | AP2S6T0 | x | X |
| Subject 3 | 3.5 | AP1S3T3.5 | √ | √ | Subject 6 | 3 | AP2S6T3 | x | √ |
| Subject 6 | 2.5 | AP1S6T2.5 | √ | √ | Subject 6 | 2.5 | AP2S6T2.5 | x | √ |
| Subject 5 | 0 | AP1S5T0 | √ | X | Subject 6 | 3.5 | AP2S6T3.5 | x | √ |
| Pool Quality Control |  | AP1PQC11 | √ | X | Pool Quality Control |  | AP2PQC11 | X | √ |

Note: Pool quality control sample is mixture of all subject’s plasma samples in equal proportion.

(ii)

| **AP 1000mg Urine sample** | | | **Run Acceptance** | | **AP 2000mg Urine sample** | | | | **Run Acceptance** | | |
| --- | --- | --- | --- | --- | --- | --- | --- | --- | --- | --- | --- |
| **Sample name** | **Time** | **Vial label** | **+ve** | **-ve** | **Sample name** | **Time** | **Vial label** | **+ve** | | **-ve** |  |
| Pooled QC |  | AP1PQCU1 | √ | √ | Pooled QC |  | AP2PQCU1 | √ | | √ |  |
| Pooled QC |  | AP1PQCU2 | √ | √ | Pooled QC |  | AP2PQCU2 | √ | | √ |  |
| Pooled QC |  | AP1PQCU3 | √ | √ | Pooled QC |  | AP2PQCU3 | √ | | √ |  |
| Pooled QC |  | AP1PQCU4 | √ | √ | Pooled QC |  | AP2PQCU4 | √ | | √ |  |
| Pooled QC |  | AP1PQCU5 | √ | √ | Pooled QC |  | AP2PQCU5 | √ | | √ |  |
| Subject 1 | 8-12 hr | AP1S1U3 | √ | √ | Subject 2 | 8-12 hr | AP2S2U3 | √ | | √ |  |
| Subject 1 | 4-8 hr | AP1S1U2 | √ | √ | Subject 1 | 4-8 hr | AP2S1U2 | √ | | √ |  |
| Subject 1 | 0-4 hr | AP1S1U1 | √ | √ | Subject 5 | 8-12 hr | AP2S5U3 | √ | | √ |  |
| Subject 1 | 0 hr | AP1S1U0 | √ | √ | Subject 6 | 4-8 hr | AP2S6U2 | √ | | √ |  |
| Pooled QC |  | PQC | √ | √ | Pooled QC |  | AP2PQCU6 | √ | | √ |  |
| Subject 2 | 0 hr | AP1S2U0 | √ | √ | Subject 3 | 0 hr | AP2S3U0 | √ | | √ |  |
| Subject 2 | 0-4 hr | AP1S2U1 | √ | √ | Subject 2 | 0 hr | AP2S2U0 | √ | | √ |  |
| Subject 2 | 4-8 hr | AP1S2U2 | √ | √ | Subject 1 | 8-12 hr | AP2S1U3 | √ | | √ |  |
| Subject 2 | 8-12 hr | AP1S2U3 | √ | √ | Subject 5 | 4-8 hr | AP2S5U2 | √ | | √ |  |
| Pooled QC |  | PQC | √ | √ | Pooled QC |  | AP2PQCU7 | √ | | √ |  |
| Subject 3 | 8-12 hr | AP1S3U3 | √ | √ | Subject 1 | 0 hr | AP2S1U0 | √ | | √ |  |
| Subject 3 | 4-8 hr | AP1S3U2 | √ | √ | Subject 4 | 0-4 hr | AP2S4U1 | √ | | √ |  |
| Subject 3 | 0-4 hr | AP1S3U1 | √ | √ | Subject 2 | 4-8 hr | AP2S2U2 | √ | | √ |  |
| Subject 3 | 0 hr | AP1S3U0 | √ | √ | Subject 5 | 0-4 hr | AP2S5U1 | √ | | √ |  |
| Pooled QC |  | PQC | √ | √ | Pooled QC |  | AP2PQCU8 | √ | | √ |  |
| Subject 4 | 0 hr | AP1S4U0 | √ | √ | Subject 6 | 8-12 hr | AP2S6U3 | √ | | √ |  |
| Subject 4 | 0-4 hr | AP1S4U1 | √ | √ | Subject 5 | 0 hr | AP2S5U0 | √ | | √ |  |
| Subject 4 | 4-8 hr | AP1S4U2 | √ | √ | Subject 6 | 0-4 hr | AP2S6U1 | √ | | √ |  |
| Subject 4 | 8-12 hr | AP1S4U3 | √ | √ | Subject 4 | 0 hr | AP2S4U0 | √ | | √ |  |
| Pooled QC |  | PQC | √ | √ | Pooled QC |  | AP2PQCU9 | √ | | √ |  |
| Subject 5 | 8-12 hr | AP1S5U3 | X* | √ | Subject 3 | 4-8 hr | AP2S3U2 | √ | | √ |  |
| Subject 5 | 4-8 hr | AP1S5U2 | X | √ | Subject 3 | 8-12 hr | AP2S3U3 | √ | | √ |  |
| Subject 5 | 0-4 hr | AP1S5U1 | X | √ | Subject 2 | 0-4 hr | AP2S2U1 | √ | | √ |  |
| Subject 5 | 0 hr | AP1S5U0 | X | √ | Subject 4 | 4-8 hr | AP2S4U2 | √ | | √ |  |
| Pooled QC |  | PQC | X | √ | Pooled QC |  | AP2PQCU10 | √ | | √ |  |
| Subject 6 | 0 hr | AP1S6U0 | X | √ | Subject 1 | 0-4 hr | AP2S1U1 | √ | | √ |  |
| Subject 6 | 0-4 hr | AP1S6U1 | X | √ | Subject 6 | 0 hr | AP2S6U0 | √ | | √ |  |
| Subject 6 | 4-8 hr | AP1S6U2 | X | √ | Subject 4 | 8-12 hr | AP2S4U3 | √ | | √ |  |
| Subject 6 | 8-12 hr | AP1S6U3 | X | √ | Subject 3 | 0-4 hr | AP2S3U1 | √ | | √ |  |
| Pooled QC |  | AP1PQCU11 | X | √ | Pooled QC |  | AP2PQCU11 | √ | | √ |  |

Note: Pool quality control sample is mixture of all subject’s urine samples in equal proportion. * Instrument restart.

Table S4. Individual subject plasma concentrations of (i) andrographolide, (ii) 14-deoxyandrographolide, and (iii) neoandrographolide following administration of 2000 mg Andrographis paniculata capsules.

(i)

| **Time** | **Andrographolide concentration (ng/ml)** | | | | | **Mean concentration (ng/ml)** |
| --- | --- | --- | --- | --- | --- | --- |
|  | S1 | S2 | S3 | S4 | S5 |  |
| 0.00 | 0.00 | 0.00 | 0.00 | 0.00 | 0.00 | 0 |
| 0.50 | 3.76 | 0.00 | 39.35 | 0.00 | 0.00 | 8.622 |
| 1.00 | 2.41 | 3.98 | 13.78 | 18.33 | 0.00 | 7.6996 |
| 1.50 | 6.36 | 5.56 | 14.17 | 22.12 | 2.52 | 10.145 |
| 2.00 | 5.31 | 5.07 | 4.19 | 12.72 | 3.77 | 6.2104 |
| 2.50 | 2.17 | 3.13 | 3.47 | 5.06 | 3.17 | 3.3999 |
| 3.00 | 3.86 | 4.68 | 0.00 | 4.20 | 2.38 | 3.0233 |
| 3.50 | 2.81 | 0.00 | 0.00 | 2.09 | 1.59 | 1.2987 |
| 4.00 | 0.00 | 0.00 | 0.00 | 0.00 | 0.54 | 0.1073 |
| 5.00 | 0.00 | 0.00 | 0.00 | 0.00 | 1.62 | 0.3249 |
| 6.00 | 0.00 | 0.00 | 0.00 | 0.00 | 1.12 | 0.2247 |
| 8.00 | 0.00 | 0.00 | 0.00 | 0.00 | 0.00 | 0 |
| 10.00 | 0.00 | 0.00 | 0.00 | 0.00 | 0.00 | 0 |
| 12.00 | 0.00 | 0.00 | 0.00 | 0.00 | 0.00 | 0 |
| 24.00 | 0.00 | 0.00 | 0.00 | 0.00 | 0.00 | 0 |

(ii)

| **Time** | **14 deoxyandrographolide concentration (ng/ml)** | | | | | **Mean concentration (ng/ml)** |
| --- | --- | --- | --- | --- | --- | --- |
|  | S1 | S2 | S3 | S4 | S5 |  |
| 0.00 | 0.00 | 0.00 | 0.00 | 0.00 | 0.00 | 0.00 |
| 0.50 | 4.67 | 0.00 | 23.39 | 1.49 | 3.04 | 6.52 |
| 1.00 | 2.88 | 4.36 | 10.13 | 10.02 | 2.29 | 5.94 |
| 1.50 | 7.71 | 4.51 | 6.33 | 13.19 | 3.39 | 7.02 |
| 2.00 | 5.18 | 3.40 | 3.06 | 4.70 | 4.99 | 4.27 |
| 2.50 | 3.32 | 2.73 | 2.52 | 5.46 | 3.66 | 3.54 |
| 3.00 | 6.06 | 4.33 | 1.77 | 1.92 | 3.28 | 3.47 |
| 3.50 | 3.78 | 0.00 | 0.00 | 2.29 | 2.09 | 1.63 |
| 4.00 | 0.00 | 0.00 | 0.00 | 1.69 | 1.36 | 0.61 |
| 5.00 | 0.00 | 0.00 | 2.59 | 1.80 | 2.40 | 1.36 |
| 6.00 | 0.00 | 0.00 | 0.00 | 2.01 | 1.70 | 0.74 |
| 8.00 | 0.00 | 0.00 | 0.00 | 2.47 | 0.00 | 0.49 |
| 10.00 | 0.00 | 0.00 | 0.00 | 0.00 | 0.00 | 0.00 |
| 12.00 | 0.00 | 0.00 | 0.00 | 0.00 | 0.00 | 0.00 |
| 24.00 | 0.00 | 0.00 | 0.00 | 0.00 | 0.00 | 0.00 |

(iii)

| **Time** | **Neoandrographolide concentration (ng/ml)** | | | | | **Mean concentration (ng/ml)** |
| --- | --- | --- | --- | --- | --- | --- |
|  | S1 | S2 | S3 | S4 | S5 |  |
| 0.00 | 0.00 | 0.00 | 0.00 | 0.00 | 0.00 | 0.00 |
| 0.50 | 13.25 | 0.77 | 82.88 | 1.90 | 15.38 | 22.84 |
| 1.00 | 21.32 | 45.33 | 75.58 | 41.13 | 27.09 | 42.09 |
| 1.50 | 41.44 | 43.74 | 49.96 | 109.10 | 48.03 | 58.45 |
| 2.00 | 32.00 | 28.01 | 24.32 | 78.17 | 86.82 | 49.86 |
| 2.50 | 19.68 | 21.74 | 13.08 | 53.88 | 65.85 | 34.85 |
| 3.00 | 24.06 | 36.51 | 7.05 | 43.85 | 59.49 | 34.19 |
| 3.50 | 22.19 | 15.12 | 0.00 | 33.53 | 31.05 | 20.38 |
| 4.00 | 6.67 | 12.85 | 0.00 | 21.95 | 18.46 | 11.99 |
| 5.00 | 11.26 | 16.17 | 0.00 | 20.59 | 9.61 | 11.53 |
| 6.00 | 4.57 | 5.29 | 0.00 | 11.91 | 4.65 | 5.28 |
| 8.00 | 0.97 | 1.69 | 0.00 | 2.08 | 1.35 | 1.22 |
| 10.00 | 0.00 | 0.00 | 0.00 | 0.00 | 0.00 | 0.00 |
| 12.00 | 0.00 | 0.00 | 0.00 | 0.00 | 0.00 | 0.00 |
| 24.00 | 0.00 | 0.00 | 0.00 | 0.00 | 0.00 | 0.00 |
