# Supplementary material for: Clinical pharmacokinetics and pharmacometabolomics of Andrographis paniculata capsules: Bridging drug disposition and metabolic response to precision medicine
Source: Naunyn Schmiedebergs Arch Pharmacol. 2025 Oct 2;399(3):3561–73. doi: 10.1007/s00210-025-04656-0 (PMC12935787; doi:10.1007/s00210-025-04656-0)
Supplement: Supplementary file 2 — Supplementary file2 (PDF 49 KB) [file 210_2025_4656_MOESM2_ESM.pdf]

Minimum reporting standards document – reporting the use of different QC samples in untargeted studies (version 1)

Authors: Metabolomics Quality Assurance & Quality Control Consortium (mQACC) Community Engagement Working group

1. Please complete this form for each different analytical platform applied in the reported study. Different LC-MS assays can be grouped together in to a single form. Different NMR assays can be grouped together in to a single form.
2. If multiple different types of QC samples are used then list all in this form.
3. Definitions for different QC sample types is available at the end of the document

Questions

Q1. Which analytical platform(s) was applied in this study?

- NMR spectroscopy ☐
- GC-MS ☐
- LC-MS ☑
- CE-MS ☐
- DIMS ☐
- IR/Raman spectroscopy ☐
- Other ☐

Q2. Was a system suitability sample or suitability sample used during the reported study and is its composition and acceptance criteria reported in the manuscript?

YES ☑ manuscript section 2.3.2.

NO ☐

NOT APPLICABLE ☐

Q3. Were internal standards used during the reported study and are the composition and acceptance criteria reported in the manuscript? For NMR, was an alternative calibration method, such as ERECTIC, used?

YES ☑ Gliclazide. Supplementary Figure S6 & S7.

NO ☐

NOT APPLICABLE ☐

Q4. Were blank samples used during the reported study and are the composition and acceptance criteria reported in the manuscript?

YES ☑ Blank water, blank plasma, blank urine (with/without IS). Supplementary Figure S6 & S7.

NO ☐

Q5. Were one or multiple types of pooled QC samples used during the reported study and are the composition and acceptance criteria reported in the manuscript?

YES ☑ Supplementary Figure S8.

NO ☐

NOT APPLICABLE

Q6. Were sample collection, storage and thawing processes reported in the manuscript?

YES ☑ Manuscript section 2.3.2.

NO ☐

Q7. Were the order for sample preparation and data collection of biological samples randomised and is this reported in the manuscript?

YES ☑ Supplementary Table S3.

NO ☐

Q8. Are the QC sample data available in a metabolomics data repository (e.g. MetaboLights or Metabolomics Workbench)?

YES ☐

NO ☑

NOT APPLICABLE ☐

Definitions

1. A ***system suitability test (SST)*** sample is a solution containing a small number of authentic chemical standards (typically five to ten analytes) from which the acquired data can be quickly assessed for accuracy and precision in an automated computational approach. For LC-MS and
